# Supplementary material for: An RNA Repair Operon Regulated by Damaged tRNAs
Source: Cell Rep. Author manuscript; Available in PMC 2021 Jan 7. (PMC7790460; doi:10.1016/j.celrep.2020.108527)
Supplement: 1 [file NIHMS1657604-supplement-1.pdf]

Cell Reports, Volume *33*

Supplemental Information

An RNA Repair Operon

Regulated by Damaged tRNAs

Kevin J. Hughes, Xinguo Chen, A. Maxwell Burroughs, L. Aravind, and Sandra L. Wolin

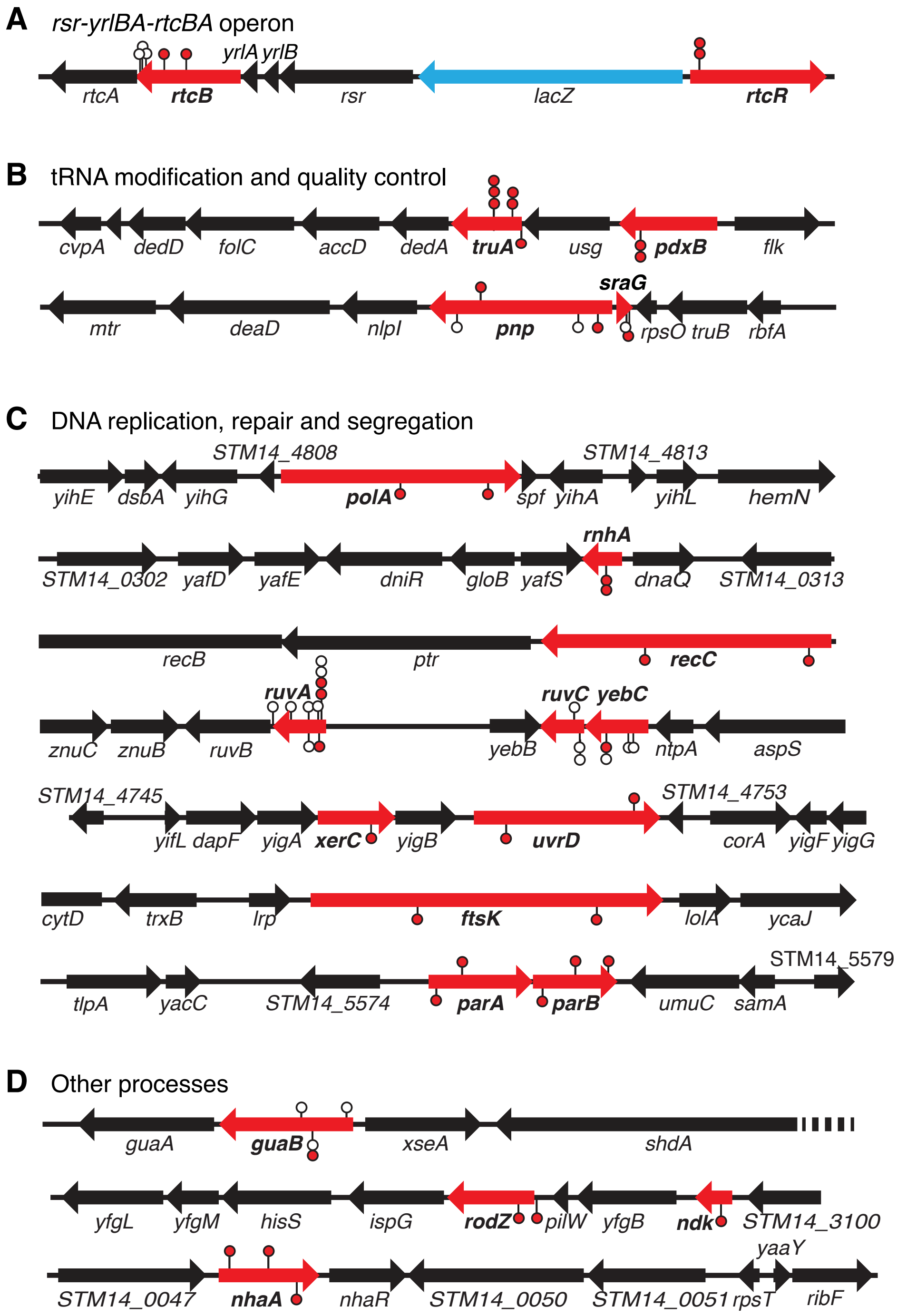

Figure S1, related to Figure 1. Location and orientation of transposon insertions resulting in P_{rsr}-lacZ expression.

(A-D) Transposon insertion sites in *S.* Typhimurium 14028s (red circles) and SL1344 (white circles) strains are indicated. Insertions in the Watson strand are shown above each locus and insertions in the Crick strand are shown below. Loci containing at least two independent insertions are shown, together with *xerC* and *ndk*, which are upstream from genes containing multiple insertions. Loci not described in the main text are *pdxB*, which encodes erythronate-4-phosphate dehyrogenate, which functions in the synthesis of pyridoxal 5′-phosphate, *xerC*, a site-specific DNA recombinase required for chromosome segregation (Blakely et al., 1993) (panel C), *guaB*, which encodes the inosine monophosphate dehydrogenase that functions in guanine biosynthesis, *ndk*, which encodes nucleotide diphosphate kinase, *rodZ*, a transmembrane protein required for the *E. coli* rod shape (Shiomi et al., 2008) and *nhaA*, which encodes the N+/H+ antiporter that maintains intracellular pH (Padan et al., 2005) (all panel D). The insertion in *pdxB* could have a polar effect on *truA* transcription.

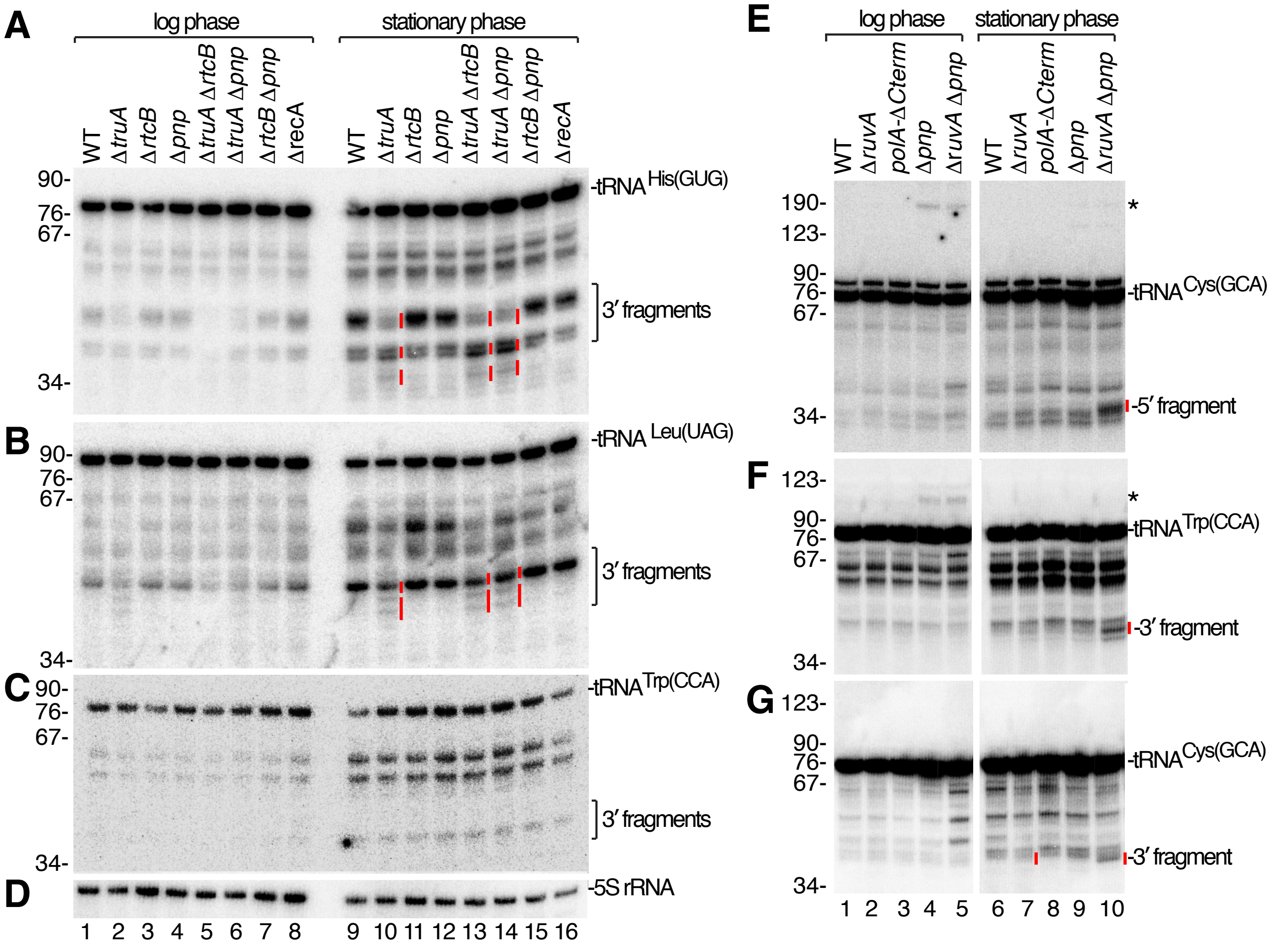

Figure S2, related to Figure 2. Accumulation of tRNA fragments in mutant strains.

(A-D) Following growth to mid-log (lanes 1-8) or stationary phase (lanes 9-16), RNA was extracted from the indicated strains and subjected to Northern blotting. Blots were probed to detect 3' halves of $tRNA^{His(GUG)}$ (A), $tRNA^{Leu(UAG)}$ (B) and $tRNA^{Trp(CCA)}$ (C). Fragments that differ in mobility or levels in Δ*truA* strains grown in stationary phase are indicated with red lines. As a loading control, the blot was reprobed to detect 5S rRNA (D).

(E-G) RNA from the indicated strains grown to mid-log (lanes 1-5) or stationary phase (lanes 6-10) was subjected to Northern blotting to detect 5' halves of $tRNA^{Cys(GCA)}$ (E) and 3' halves of $tRNA^{Trp(CCA)}$ (F) and $tRNA^{Cys(GCA)}$ (G). The asterisks denote tRNA precursors that accumulate in Δ*pnp* strains. Fragments differing in mobility or levels in mutant strains grown in stationary phase are indicated with red lines.

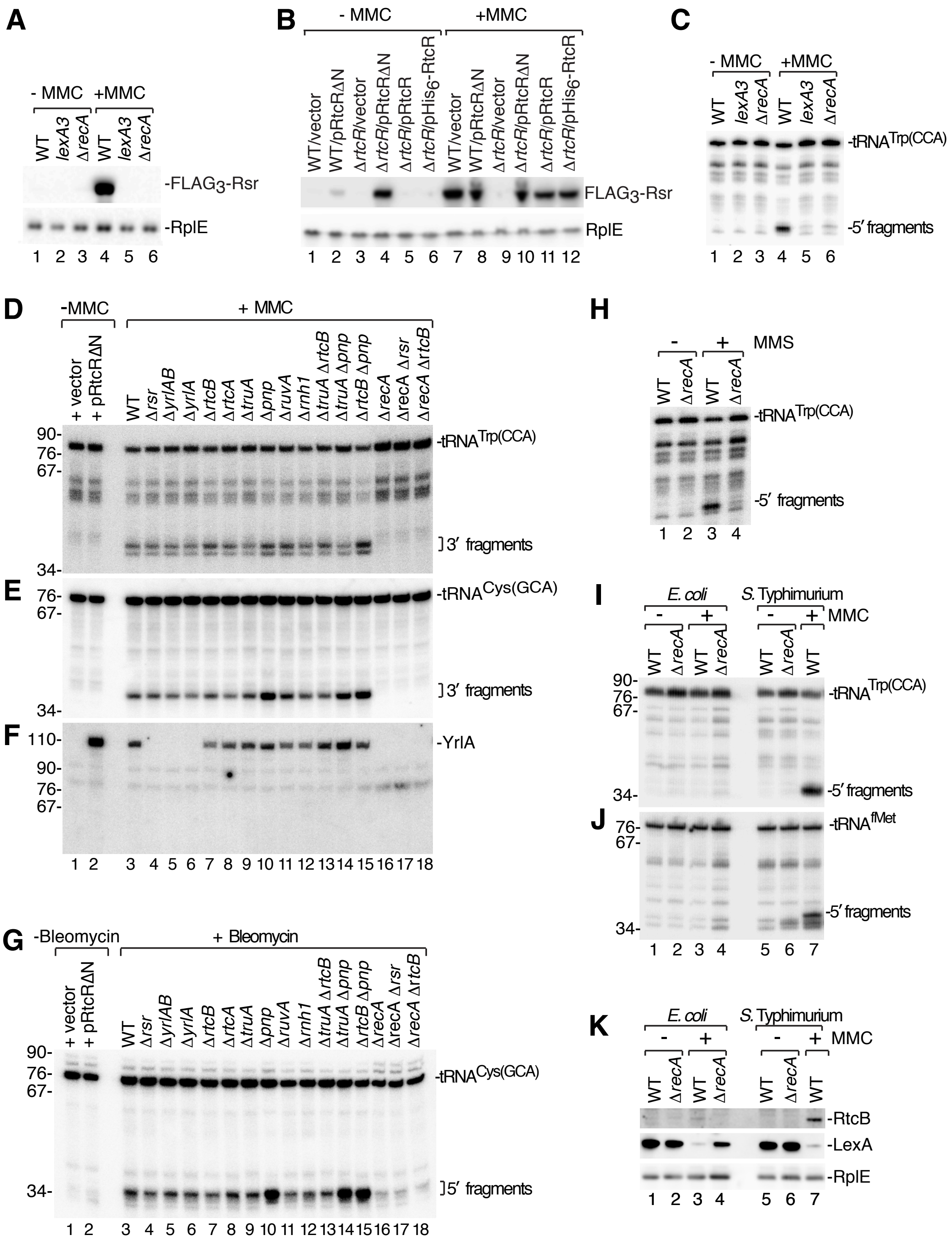

Figure S3, related to Figure 4. DNA damage results in tRNA cleavage and operon induction

(A) After treating wild-type, Δ*recA* and *lexA3* strains carrying *FLAG$_3$-rsr* without (lanes 1-3) or with MMC (lanes 4-6) for 2 hours, lysates were immunoblotted to detect FLAG$_3$-Rsr and RplE (loading control).

(B) Wild-type (lanes 1, 2, 7, 8) and Δ*rtcR* strains (lanes 3-6 and 9-12) carrying *FLAG$_3$-rsr* were grown without (lanes 1-6) or with MMC (lanes 7-12) and the resulting lysates subjected to Western blotting to detect FLAG$_3$-Rsr and Rpl5. To confirm that lack of operon expression was due to loss of RtcR, Δ*rtcR* strains carrying plasmids expressing RtcR (lanes 5, 11) and His$_6$-RtcR (lanes 6, 12) were examined. Strains carrying pRtcRΔN were also assayed (lanes 4, 10).

(C) After treating the strains without (A) (lanes 1-3) or with MMC (lanes 4-6) for 2 hours, RNA was extracted and subjected to Northern blotting to detect $tRNA^{Trp(CCA)}$ 5' fragments.

(D-F) After two hours in MMC, RNA was extracted from the indicated strains and subjected to Northern analysis to detect 3' fragments of $tRNA^{Trp(CCA)}$ (D), $tRNA^{Cys(GCA)}$ (E) or 5' fragments of YrlA RNA (F) (lanes 3-18). As a control, RNA from wild-type cells carrying the pRtcRΔN plasmid or an empty vector was assayed (lanes 1-2).

(G) After growing the indicated strains to mid-log, bleomycin was added and the strains incubated for 2 hours at 37°C. RNA was subjected to Northern analysis to detect 5' fragments of $tRNA^{Cys(GCA)}$ (lanes 3-18). Lanes 1-2, RNA from wild-type cells carrying either an empty vector or the pRtcRΔN plasmid.

(H) After treating wild-type and Δ*recA FLAG*$_3$*-rsr* strains either without (lanes 1-2) or with MMS (lanes 3-4) for 2 hours, RNA was subjected to Northern blotting to detect 5' halves of $tRNA^{Trp(CCA)}$.

(I and J) After growing *E. coli* MG1655 and *S*. Typhimurium 14028s strains with or without MMC, RNA was probed to detect 5' fragments of $tRNA^{Trp(CCA)}$ (I) and $tRNA^{fMet}$ (J).

(K) After growth with or without MMC for two hours, lysates of the indicated *E. coli* and *S*. Typhimurium strains were subjected to immunoblotting to detect RtcB, LexA and RplE.

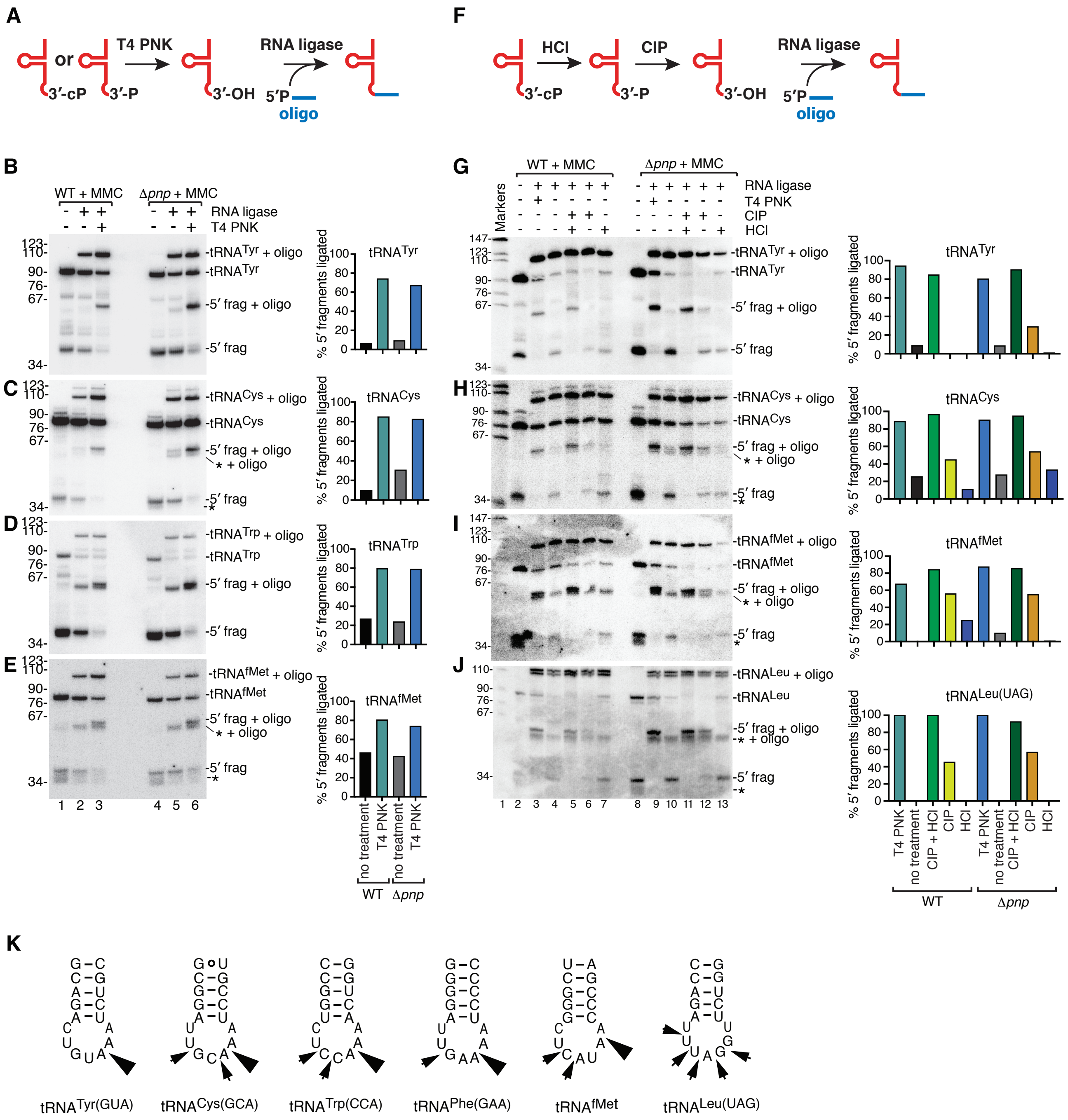

Figure S4, related to Figure 5. tRNA 5' fragments end in cyclic phosphate

(A) Schematic of strategy used to distinguish 5' fragments ending in 2', 3'-cyclic phosphate and 3'-phosphate from 5' fragments ending in 3'-OH.

(B-E) Left panels: After growing wild-type (lanes 1-3) and Δ*pnp* strains (lanes 4-6) with MMC for two hours, RNA was extracted and incubated without (lanes 1, 2, 4, 5) or with (lanes 3, 6) T4 PNK prior to adding T4 RNA ligase and a 5'-phosphate-containing oligonucleotide. After gel fractionation, Northern blotting was used to detect $tRNA^{Tyr(GUA)}$ (B), $tRNA^{Cys(GCA)}$ (C), $tRNA^{Trp(CCA)}$ (D) and $tRNA^{fMet}$ (E). Right panels: Quantitation. The asterisks in C and E denote shorter fragments of $tRNA^{Cys(GCA)}$ and $tRNA^{fMet}$ that end in 3'-OH.

(F) Schematic of strategy used to distinguish 5' fragments ending in 2', 3'-cyclic phosphate from 5' fragments ending in 3'-phosphate.

(G-J) To distinguish between 3'-phosphate and 2', 3'-cyclic phosphate, RNA from MMC-treated wild-type (lanes 2-7) and Δ*pnp* strains (lanes 8-13) was incubated with acid (HCl) and calf-intestinal phosphatase (CIP, lanes 5 and 11) or CIP alone (lanes 6 and 12), prior to adding T4 RNA ligase and the 5'-phosphate containing oligonucleotide. Northern blotting was used to detect $tRNA^{Tyr(GUA)}$ (G), $tRNA^{Cys(GCA)}$ (H), $tRNA^{fMet}$ (I) and $tRNA^{Leu(UAG)}$ (J). As a positive control, T4 PNK was added in place of CIP (lanes 3 and 9). As negative controls, the indicated components were omitted from the reactions (lanes 2, 4, 7, 8, 10, 13). Lane 1, size markers. Right panels: Quantitation. The asterisks in H, I and J denote shorter fragments of $tRNA^{Cys(GCA)}$, $tRNA^{fMet}$ and $tRNA^{Leu(UAG)}$ that end in 3'-OH.

(K) Cleavage sites of $tRNA^{Tyr(GUA)}$, $tRNA^{Cys(GCA)}$, $tRNA^{Trp(CCA)}$, $tRNA^{Phe(GAA)}$, $tRNA^{fMet}$ and $tRNA^{Leu(UAG)}$ determined by 3' RACE. For each tRNA, at least 9 cDNAs were sequenced (see Table S2). Arrowheads denote 3' ends present in more than half the sequences, while small arrows denote minor 3' ends.

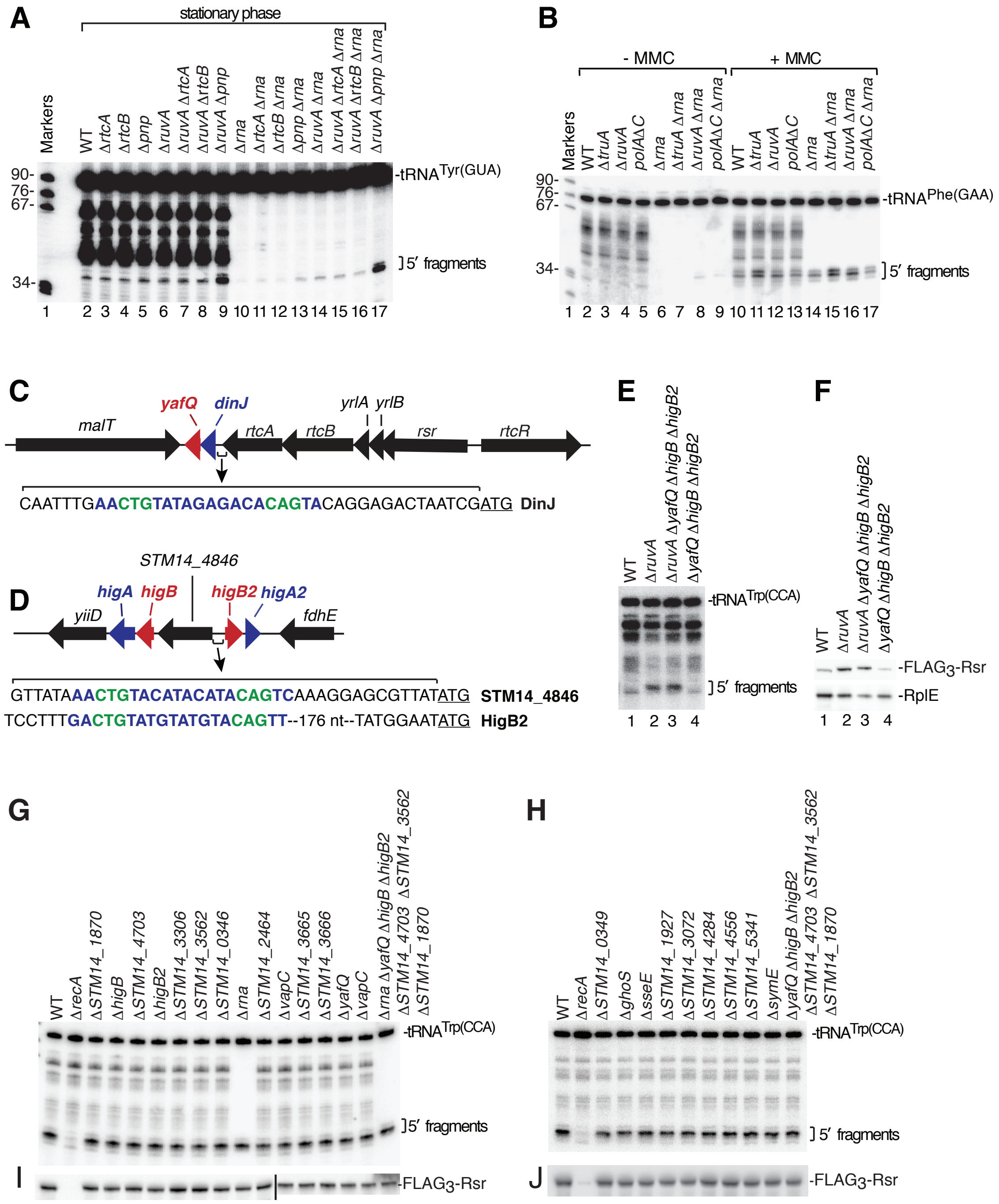

Figure S5, related to Figure 5. Deletion of multiple candidate endonucleases does not affect tRNA cleavage or operon activation

(A) RNA from the experiment shown in Figures 5A-5C was subjected to Northern blotting to detect 5' fragments of tRNA$^{Tyr\ (GUA)}$.

(B) RNA from the experiment shown in Figures 5D-G was subjected to Northern blotting to detect 5' fragments of tRNA$^{Phe(GAA)}$.

(C and D) Genetic map of the *dinJ-yafQ* (C) and *higB-higA* and *higB2-higA2* (D) neighborhoods. The sequences of the putative LexA sites are shown.

(E) RNA extracted from the indicated strains was subjected to Northern blotting to detect 5' fragments of tRNA$^{Trp(CCA)}$.

(F) Lysates of the strains in (E) were subjected to immunoblotting to detect $FLAG_3$-Rsr and RplE (loading control).

(G and H) After growing for two hours in MMC, RNA was isolated from the indicated strains and subjected to Northern blotting to detect $tRNA^{Trp(CCA)}$ 5' fragments. In the last lanes of (G) and (H), RNA was extracted from strains lacking seven (G) or six (H) potential nucleases.

(I and J) After two hours of growth in MMC, lysates were prepared from the strains shown in (G and H) and immunoblotting was performed to detect $FLAG_3$-Rsr.

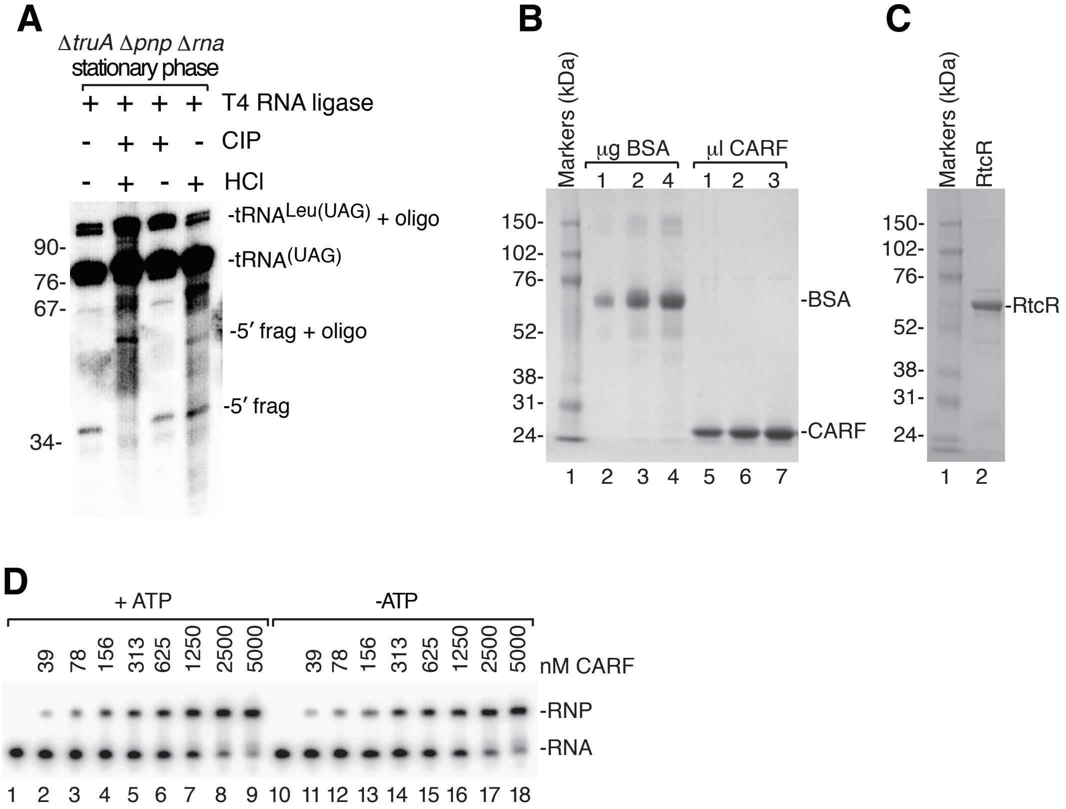

Figure S6, related to Figure 6. Binding of 5' tRNA halves to the RtcR CARF domain

(A) To determine whether the 5' tRNA$^{Leu(UAG)}$ fragments that accumulate in Δ*truA* Δ*pnp* Δ*rna* strains in stationary phase ended with 3'-OH, 3'-phosphate, or 2', 3'-cyclic phosphate, total RNA from these cells was incubated with acid (HCl) and or calf intestinal phosphatase (CIP) prior to adding T4 RNA ligase and the 5'-phosphate containing oligonucleotide. RNA in lane 1 received no acid or CIP. Northern blotting was used to detect tRNA$^{Leu(UAG)}$.

(B) To quantitate the purified protein, increasing amounts of CARF domain were subjected to SDS-PAGE together with 1, 2 and 4 μg of purified bovine serum albumin and visualized by Coomassie blue staining.

(C) Purified RtcR (1 μg) was subjected to SDS-PAGE and stained with Coomassie blue.

(D) To examine whether binding to the isolated CARF domain was dependent on ATP, 5' tRNA$^{Leu(UAG)}$ halves ending in cyclic phosphate were mixed with the indicated concentrations of the isolated CARF domain with or without 1 mM ATP.

Table S2. 3' ends of tRNA 5' halves that accumulate in MMC (determined by 3' RACE), related to Figures 5 and S4.

tRNA$^{Cys(GCA)}$	
**GCGGATTGCAAATCCGTCTAGTCCGGTTCGACTCCGGAACGCGCCTCCA**	
GCGGATTGCA	(8)
GCGGATTGC	(1)
GCGGATT	(3)
tRNA$^{Trp(CCA)}$	
**CCGGTCTCCAAAACCGGGTGTTGGGAGTTCGAGTCTCTCCGCCCCTGCCA**	
CCGGTCTCCA	(6)
CCGGTCTC	(2)
CCGGTCT	(1)
tRNA$^{Tyr(GUA)}$	
**GCAGACTGTAAATCTGCCGTCATCGACTTCGAAGGTTCGAATCCTTCCCCCACCACCA**	
GCAGACTGTA	(12)
tRNA$^{Phe(GAA)}$	
**GGGGATTGAAAATCCCCGTGTCCTTGGTTCGATTCCGAGTCCGGGCACCA**	
GGGGATTGAA	(10)
GGGGATT	(2)
tRNA$^{(fMet)}$	
**TCGGGCTCATAACCCGAAGGTCGTCGGTTCAAATCCGGCCCCCGCAACCA**	
TCGGGCTCATA	(8)
TCGGGCTC	(4)
TCGGGCT	(2)
tRNA$^{Leu(UAG)}$	
**CCAGATTTAGGTTCTGGCGCCGCAAGGTGTGCGAGTTCAAGTCTCGCCTCCCGCACCA**	
CCAGATTTAG	(1)
CCAGATTTA	(4)
CCAGATTT	(3)
CCAGATT	(5)
CCAGAT	(2)

Sequences of clones obtained from 3' RACE. The number in parentheses denotes the number of independent clones for each sequence. A portion of the full length tRNA sequence is at the top in bold and the anticodon is underlined.
