# Supplementary material for: An RNA Repair Operon Regulated by Damaged tRNAs
Source: Cell Rep. Author manuscript; Available in PMC 2021 Jan 7. (PMC7790460; doi:10.1016/j.celrep.2020.108527)
Supplement: 4 [file NIHMS1657604-supplement-4.pdf]

Cell Reports

Article

An RNA Repair Operon Regulated by Damaged tRNAs

Graphical Abstract

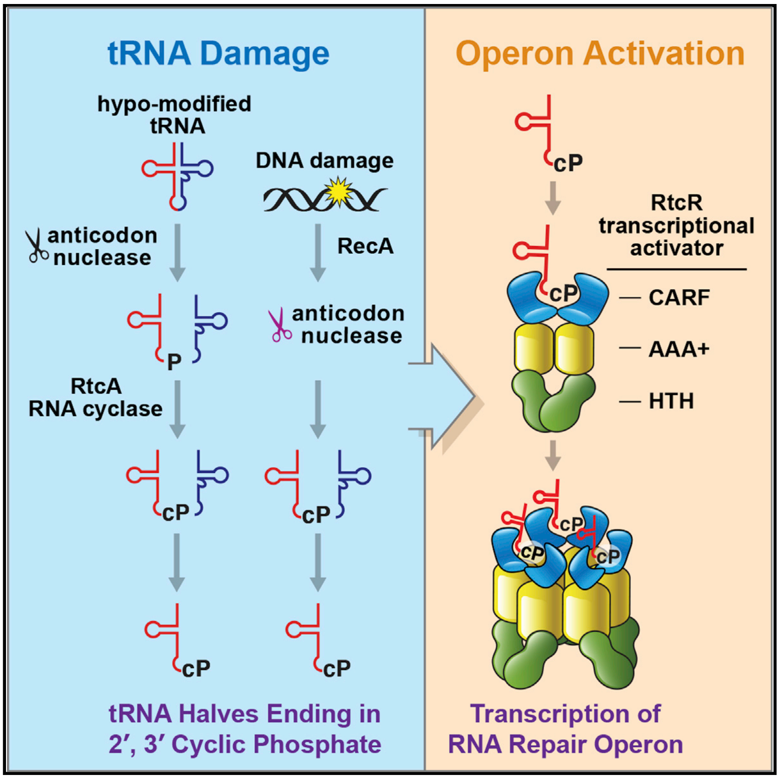

Authors

Kevin J. Hughes, Xinguo Chen, A. Maxwell Burroughs, L. Aravind, Sandra L. Wolin

Correspondence

xinguo.chen@nih.gov (X.C.), sandra.wolin@nih.gov (S.L.W.)

In Brief

Hughes et al. demonstrate that a bacterial RNA repair operon, containing the RtcB RNA ligase and the RtcA RNA cyclase, is regulated by binding of 5′ tRNA halves ending in 2′, 3′-cyclic phosphate to the RtcR transcriptional activator. These studies show how tRNA fragments can regulate bacterial gene expression.

Highlights

- An RNA repair operon is activated by mutations that cause tRNA halves to accumulate
- Operon expression and accumulation of tRNA halves occur upon DNA damage
- The 5′ tRNA halves that accumulate end in 2′, 3′-cyclic phosphate
- The RtcR transcriptional activator oligomerizes upon binding these 5′ tRNA halves

Hughes et al., 2020, Cell Reports *33*, 108527
December 22, 2020
https://doi.org/10.1016/j.celrep.2020.108527

CellPress

Cell Reports

CellPress
OPEN ACCESS

Article

An RNA Repair Operon Regulated by Damaged tRNAs

Kevin J. Hughes,[1,2,4] Xinguo Chen,[1,4,*] A. Maxwell Burroughs,[3] L. Aravind,[3] and Sandra L. Wolin[1,5,*]
[1]RNA Biology Laboratory, Center for Cancer Research, National Cancer Institute, National Institutes of Health, Frederick, MD 21702, USA
[2]Department of Cell Biology, Yale School of Medicine, New Haven, CT 06510, USA
[3]Computational Biology Branch, National Center for Biotechnology Information, National Library of Medicine, National Institutes of Health, Bethesda, MD, 20894, USA
[4]These authors contributed equally
[5]Lead Contact
*Correspondence: xinguo.chen@nih.gov (X.C.), sandra.wolin@nih.gov (S.L.W.)
https://doi.org/10.1016/j.celrep.2020.108527

SUMMARY

Many bacteria contain an RNA repair operon, encoding the RtcB RNA ligase and the RtcA RNA cyclase, that is regulated by the RtcR transcriptional activator. Although RtcR contains a divergent version of the CARF (CRISPR-associated Rossman fold) oligonucleotide-binding regulatory domain, both the specific signal that regulates operon expression and the substrates of the encoded enzymes are unknown. We report that tRNA fragments activate operon expression. Using a genetic screen in *Salmonella enterica* serovar Typhimurium, we find that the operon is expressed in the presence of mutations that cause tRNA fragments to accumulate. RtcA, which converts RNA phosphate ends to 2′, 3′-cyclic phosphate, is also required. Operon expression and tRNA fragment accumulation also occur upon DNA damage. The CARF domain binds 5′ tRNA fragments ending in cyclic phosphate, and RtcR oligomerizes upon binding these ligands, a prerequisite for operon activation. Our studies reveal a signaling pathway involving broken tRNAs and implicate the operon in tRNA repair.

INTRODUCTION

Most organisms contain repair systems that ligate RNA fragments generated by nuclease cleavage or removal of encoded intervening sequences. A major repair pathway involves RtcB, which joins pre-tRNA halves after intron excision in metazoans and archaea. In metazoans, RtcB also ligates mRNA encoding the XBP1 transcription factor as part of the unfolded protein response (Englert et al., 2011; Popow et al., 2011; Jurkin et al., 2014; Kosmaczewski et al., 2014). RtcB joins RNA 5′-OH ends to 2′, 3′-cyclic phosphate or 3′-phosphate RNA ends (Englert et al., 2011; Tanaka et al., 2011). RtcB is also present in bacteria, where its functions are less understood. The only reported substrate is 16S rRNA, since *E. coli* RtcB can re-ligate a 3′ fragment of 16S rRNA to the rRNA body after cleavage by a stress-induced endonuclease (Temmel et al., 2017). *In vitro*, *E. coli* RtcB can add a ppG cap to DNAs ending in 3′-phosphate (Das et al., 2013); however, the role of this activity *in vivo* is unclear.

In bacteria, RtcB is often expressed as part of a highly regulated "RNA repair" operon. In this operon, *rtcB* is adjacent to *rtcA*, which encodes an RNA cyclase that converts 3′-phosphate ends to 2′, 3′-cyclic phosphate (Genschik et al., 1998; Tanaka and Shuman, 2011). In some bacteria, including *Salmonella enterica* serovar Typhimurium (*S.* Typhimurium), the *rtcBA* operon also encodes orthologs of a human RNA-binding protein known as the Ro60 autoantigen and noncoding RNAs called Y RNAs (Chen et al., 2013; Das and Shuman, 2013; Burroughs and Aravind, 2016). The functions of bacterial Ro60 proteins (called Rsr for Ro sixty-related) and Y RNAs have only been studied in *Deinococcus radiodurans*, where Rsr is tethered by Y RNA to the 3′ to 5′ exoribonuclease polynucleotide phosphorylase (PNPase), specializing the nuclease for structured RNA decay (Chen et al., 2013). Transcription of the operon is often regulated by the enhancer binding protein RtcR, which is encoded adjacent to *rtcBA* and transcribed in the opposite direction (Genschik et al., 1998).

An impediment to studying the roles of the *rtcBA* operon has been a lack of information as to how the operon is regulated. RtcR is a member of the σ^{54}-dependent enhancer binding protein family, which typically features an N-terminal signal-sensing ligand-binding domain fused to a C-terminal AAA+ ATPase domain that multimerizes and interacts with σ^{54} (Bush and Dixon, 2012). The N-terminal portion of RtcR contains a divergent form of the CARF (CRISPR-associated Rossman fold) domain (Makarova et al., 2014). Canonical CARF domains have been best characterized in type III CRISPR-Cas systems, where they are linked to effector domains such as ribonucleases. In these systems, binding of cyclic oligoadenylate (cOA) molecules to the CARF domain activates the adjacent effector (Kazlauskiene et al., 2017; Niewoehner et al., 2017). However, ligands that bind the RtcR domain have not been identified.

To determine how a *rtcBA* operon is activated, we performed a genetic screen to identify mutations that result in transcription of

This is an open access article under the CC BY license (http://creativecommons.org/licenses/by/4.0/).

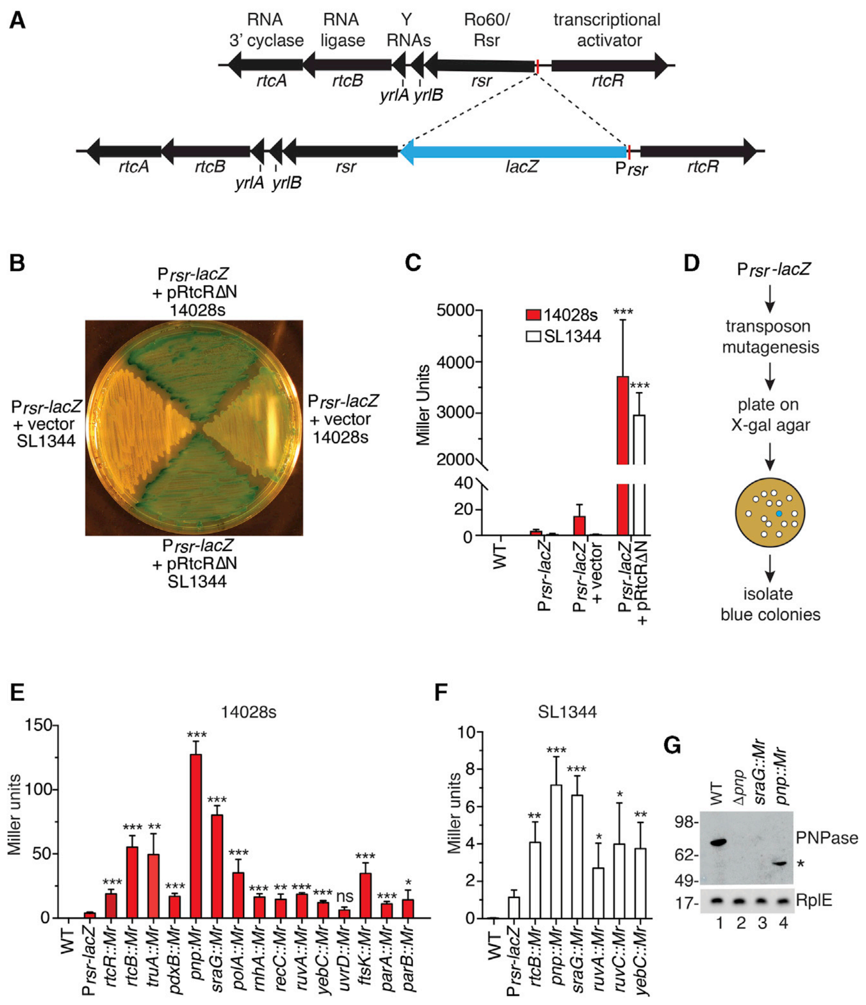

Figure 1. Identification of Mutations that Activate the Operon

(A) Map of the operon and insertion of the *lacZ* reporter behind the P*rsr* promoter.

(B) β-Galactosidase activity was visualized by growing strains on X-gal-containing agar.

(C) After growth in liquid culture, β-galactosidase was measured in P_{rsr}-*lacZ* strains carrying empty vector or pRtcRΔN.

(D) Screening strategy.

(E and F) β-Galactosidase activity was assayed in 14028s (E) and SL1344 (F) P_{rsr}-*lacZ* strains carrying the indicated transposon insertions.

(G) Immunoblotting was performed on the indicated strains to detect PNPase. Asterisk, truncated form of PNPase.

Data in (C), (E), and (F) are mean (n = 3) ± SEM. p values were calculated with two-tailed unpaired t tests relative to P_{rsr}-*lacZ* strains. *$p < 0.05$; **$p < 0.01$, and ***$p < 0.001$; ns, not significant. See also Figure S1.

the *S*. Typhimurium *rsr-yrlBA-rtcBA* operon. We report that mutations that result in accumulation of tRNA fragments activate operon expression. Some mutations result in DNA damage, and we show that a feature of the DNA damage response in *S*. Typhimurium is the activation of one or more ribonucleases that cleave tRNAs in the anticodon loop, resulting in 5′ fragments ending in 2′, 3′-cyclic phosphate. Consistent with the hypothesis that RNAs ending in 2′, 3′-cyclic phosphate are important for operon activation, overexpression of RtcA increases operon expression. We show that the RtcR CARF domain binds 5′ tRNA fragments ending in 2′, 3′-cyclic phosphate and that RtcR forms oligomers upon ligand binding. Our studies identify a signaling pathway involving tRNA and implicate the operon in the repair of damaged tRNAs.

RESULTS

Identification of Mutations that Result in Operon Activation

To identify genes that, when mutated, result in activation of the *rsr-yrlBA-rtcBA* operon, we generated *S*. Typhimurium strains in which a *lacZ* reporter was inserted upstream of *rsr* under control of the *rsr* promoter (Figure 1A). We introduced the reporter into two different virulent strains, SL1344 and 14028s, because our early experiments revealed strain-specific differences in the extent to which the operon was repressed. Specifically, while β-galactosidase was fully repressed in the SL1344 P_{rsr}-*lacZ* strain, we detected low levels of β-galactosidase in the 14028s P_{rsr}-*lacZ* strain (Figures 1B and 1C). Expression of P_{rsr}-*lacZ* was under control of the RtcR transcriptional activator, since β-galactosidase activity increased >2,500-fold in both strains when we expressed a constitutively active RtcR lacking part of the N-terminal ligand-binding domain (RtcRΔN; Genschik et al., 1998) (Figure 1C). Upon RtcRΔN expression, both P_{rsr}-*lacZ* strains also appeared strongly blue when grown on X-gal-containing agar (Figure 1B).

We used a chloramphenicol-resistant derivative of pSAM, a mariner himar1C9 transposon delivery vector (Goodman et al., 2009), to create a library of transposon insertions in the P_{rsr}-*lacZ* strains. Mutants that formed blue colonies on X-gal agar and exhibited at least a 50% increase in β-galactosidase activity compared to the parent strain were selected (Figure 1D). With these criteria, we obtained 45 mutants from 28,000 chloramphenicol-resistant 14028s P_{rsr}-*lacZ* colonies and 26 mutants from 35,000 chloramphenicol-resistant SL1344 P_{rsr}-*lacZ* colonies. Using ligation-mediated PCR, we mapped the transposon insertions to 28 distinct loci (Table S1). Six loci were identified in both strains, 17 were identified only in the 14028s strain and five only in the SL1344 strain. To confirm that the transposon insertion was responsible for the blue color, we used P22 phage transduction to transfer each transposon mutation into the parent P_{rsr}-*lacZ* strain. Loci with at least two independent transposon insertions are shown in Figure S1, together with the insertion sites (red circles, 14028s; white circles, SL1344).

Mutations in Genes Involved in tRNA Metabolism and DNA Repair Activate the Operon

The genes we identified fell into three major functional categories. One group mapped within the *rsr-yrlBA-rtcBA* operon (Figure S1A). As expected, we recovered transposon insertions that, similar to the RtcRΔN mutation, truncate the RtcR N-terminal CARF domain to render the operon constitutively active (Genschik et al., 1998). As these mutations were only recovered in the 14028s strain, our screen may not have reached saturation. We also recovered transposon insertions from both strains within *rtcB*. One explanation is that the operon is normally expressed at low levels in both strains and that decreased RtcB ligase activity results, directly or indirectly, in increased levels of ligands that activate the operon.

A second category consisted of genes with roles in tRNA metabolism (Figure S1B). Insertions in *truA*, which encodes the pseudouridine synthase that modifies uridines at positions 38, 39, and 40 in the anticodon arm of some tRNAs (Cortese et al., 1974), activate the operon, as do insertions in *pnp*, which encodes the PNPase exoribonuclease. In *E. coli*, PNPase degrades aberrant pre-tRNAs (Li et al., 2002). We also obtained mutations in *sraG*, which encodes a small RNA that regulates *pnp* mRNA levels (Fontaine et al., 2016).

Surprisingly, the largest category consisted of genes involved in DNA replication, repair, and segregation (Figure S1C). These genes included *polA*, which encodes DNA polymerase I; *rnhA*, which encodes RNase H1, which cleaves the RNA strand of RNA-DNA hybrids that form during DNA replication and transcription (Hollis and Shaban, 2011); *recC*, which encodes a subunit of the RecBCD helicase-nuclease complex that functions in double-strand break repair (Dillingham and Kowalczykowski, 2008); *ruvA* and *ruvC*, whose products resolve Holliday junctions (Wyatt and West, 2014); and *uvrD*, which encodes a helicase involved in nucleotide excision repair (Kisker et al., 2013). We also identified mutations in *yeb*C, a transcriptional regulator that contributes to *E. coli* survival after ionizing radiation (Byrne et al., 2014). Other mutations disrupted *ftsK*, which encodes a DNA translocase with roles in chromosome segregation (Kaimer and Graumann, 2011); and *parA* and *parB*, which encode proteins required for plasmid segregation (Gerdes et al., 2010). We also obtained transposon insertions in several genes that function in other processes (Figure S1D).

To compare the extent to which the various mutations increased P_{rsr}-*lacZ* expression, we performed β-galactosidase assays. We focused on the three major categories (Figures S1A–S1C) and assayed cells between the mid-logarithmic and early stationary phases of growth. For all genes that were sites of transposon insertion in both strains (*rtcB*, *pnp*, *sraG*, *ruvA*, and *yebC*), β-galactosidase levels were >10-fold higher in the 14028s strain (Figures 1E and 1F). Due to the stronger induction, subsequent experiments were performed in the 14028s strain.

As the *sraG* RNA downregulates *pnp* mRNA (Fontaine et al., 2016), it was surprising that mutations in both *pnp* and *sraG* increased operon activation. Since *sraG* overlaps the *pnp* promoter, and our mutations are within or near this promoter, we examined PNPase levels by immunoblotting. PNPase was undetectable in *sraG* mutants (Figure 1G), indicating the transposon insertions abrogate PNPase synthesis.

To confirm that loss of function of the affected genes was responsible for operon activation, we generated strains lacking open reading frames (ORFs) that were sites of transposon insertion. We monitored operon activation using western blotting to detect expression of Rsr fused at the N terminus to three copies of the FLAG epitope. When strains containing deletions in *truA*, *pnp, ruvA*, or *rnhA* were grown to mid-logarithmic phase, $FLAG_3$-Rsr increased compared to wild-type cells (Figure 2A). Although the low levels of $FLAG_3$-Rsr in Δ*rtcB* strains were not significantly different from wild-type strains, strains lacking *pnp* and either *truA* or *rtcB* contained higher levels of $FLAG_3$-Rsr than either deletion alone, revealing that deletions in PNPase, TruA, and RtcB act additively.

Since expression of the *E. coli rtcBA* operon increases in stationary phase (Temmel et al., 2017), we examined expression of the *S*. Typhimurium operon as a function of growth. Although P_{rsr}-*lacZ* expression did not change in the parental strain, expression increased more than 5-fold in the *truA* transposon mutant (*truA::Mr*) between mid-log and stationary phase (Figure 2B). Western blotting revealed that although the levels of $FLAG_3$-Rsr in wild-type cells increased less than 1.5-fold, $FLAG_3$-Rsr increased 2.8-fold in Δ*ruvA* strains, 2-fold in Δ*pnp* strains, and 5-fold in Δ*truA* strains in stationary phase relative to mid-log phase (Figure 2C). Thus, mutations that disrupt certain genes involved in tRNA metabolism and DNA repair activate expression of the *rsr-yrlBA-rtcBA* operon and the effects are more severe in stationary phase.

Distinct tRNA Fragments Accumulate in Mutant Strains

Since one category of mutations that resulted in operon activation were predicted to affect tRNA metabolism, we examined tRNA levels in the mutant strains. We examined *truA*, since pseudouridines in tRNA anticodon arms contribute to structural stability (Arnez and Steitz, 1994); *rtcB*, since RtcB ligates eukaryotic and archaeal tRNAs after intron excision; and *pnp*, which degrades aberrant pre-tRNAs (Li et al., 2002). We grew strains lacking each gene to mid-log or stationary phase, isolated RNA, and performed northern blotting to detect two tRNAs that are TruA substrates, $tRNA^{His(GUG)}$ and $tRNA^{Leu(UAG)}$. Several 5′ (Figures 2D and 2E) and 3′ (Figures S2A and S2B) fragments of these tRNAs were detected in wild-type and mutant strains during logarithmic growth and increased in cells in stationary phase.

Notably, some fragments in Δ*truA* strains were altered in mobility and/or levels compared to wild-type strains. For both $tRNA^{His(GUG)}$ and $tRNA^{Leu(UAG)}$, some 5′ and 3′ fragments were reduced in Δ*truA* strains, while others became prominent (Figures 2D, 2E, S2A, and S2B, lanes 2 and 10, red lines). Possibly, the decreased stability of these tRNAs in Δ*truA* strains renders them susceptible to cleavage at distinct sites. Fragments of tRNAs that are not TruA substrates, such as $tRNA^{Trp(CCA)}$, were unchanged in Δ*truA* strains (Figure S2C).

To determine if accumulation of specific tRNA fragments was a common feature of our mutants, we examined strains carrying deletions in genes important for DNA replication and repair. Remarkably, we detected discrete tRNA fragments in some strains. This was most apparent in stationary phase, where we detected 5′ fragments of $tRNA^{Trp(CCA)}$ and $tRNA^{fMet}$ in strains that lacked *ruvA* or were deleted for the 3′ end of the essential

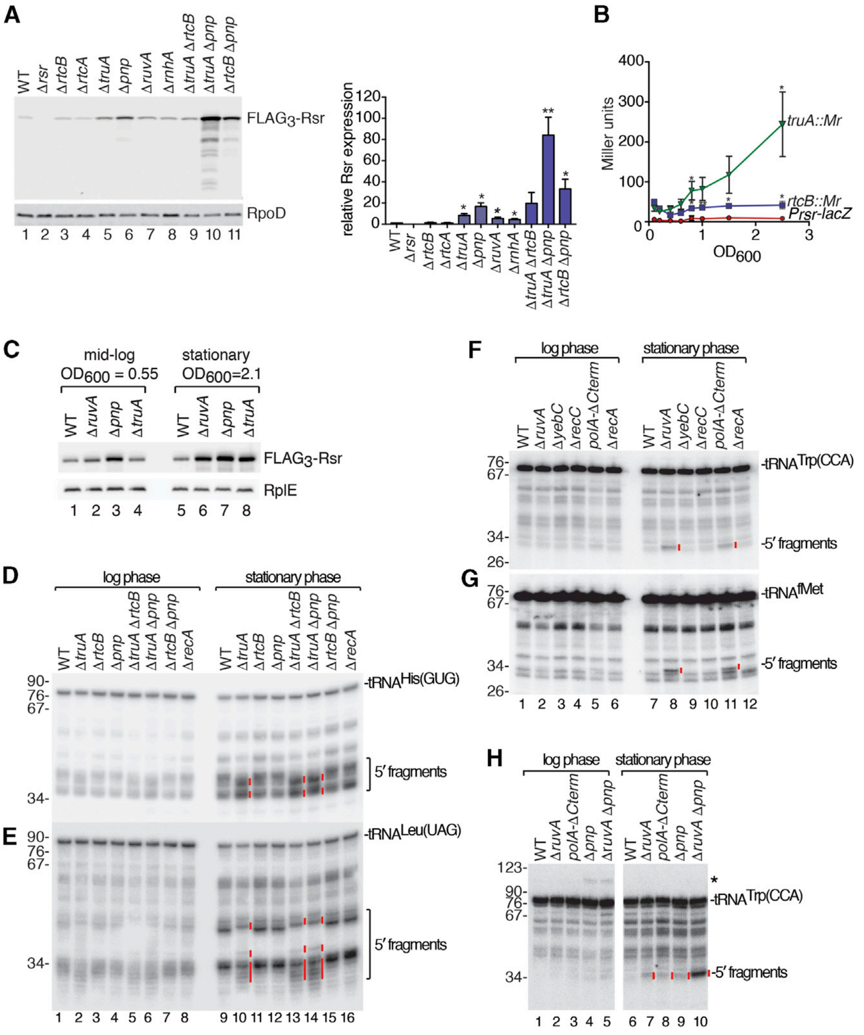

Figure 2. tRNA Fragments Accumulate in Some Mutant Strains

(A) 14028s *FLAG$_3$-rsr* strains carrying the indicated deletions were subjected to immunoblotting to detect FLAG$_3$-Rsr. RpoD, loading control. Right, quantitation (n = 3). p values are relative to the wild-type strain.

(B) β-Galactosidase activity was assayed in the indicated P$_{rsr}$-*lacZ* strains as a function of growth. p values are relative to the P$_{rsr}$-*lacZ* strain.

(C) Lysates of the indicated *FLAG$_3$-rsr* strains were subjected to immunoblotting to detect FLAG$_3$-Rsr. RplE, loading control.

(D and E) RNA from the indicated strains was subjected to northern blotting to detect 5′ halves of tRNA$^{His(GUG)}$ (D) and tRNA$^{Leu(UAG)}$ (E).

(F and G) RNA from the indicated strains was subjected to northern blotting to detect 5′ halves of tRNA$^{Trp(CCA)}$ (F) and tRNA^{fMet} (G).

(H) RNA from the indicated strains was probed to detect 5′ halves of tRNA$^{Trp(CCA)}$. Asterisk denotes a tRNA precursor.

Data in (A) and (B) are mean (n = 3) ± SEM. p values were calculated with two-tailed unpaired t tests. *p < 0.05 and **p < 0.01.

In (D)–(H), red lines denote fragments unique to mutant strains in stationary phase. See also Figure S2.

in Δ*ruvA* cells were strongly reduced in Δ*ruvA* Δ*recA* cells (Figure 3A). Western blotting revealed that although the operon was activated in Δ*ruvA* stationary phase cells, it was expressed similarly to wild-type cells in Δ*ruvA* Δ*recA* cells (Figure 3B).

In contrast, accumulation of tRNA fragments in Δ*truA* cells was unaffected by *recA* deletion (Figures 3C and 3D), supporting the hypothesis that tRNA breakage

polA gene (*polA*ΔC) (Figures 2F and 2G). These fragments, as well as 5′ and 3′ fragments of other tRNAs, such as tRNA$^{Cys(GCA)}$, increased further in Δ*ruvA* cells that also lacked *pnp* (Figures 2H and S2E–S2G), indicating that PNPase may degrade the fragments. Although we did not detect specific fragments in Δ*yebC* or Δ*recC* strains, low levels of these fragments may be obscured by the background of nonspecific fragments that we show later to be irrelevant for operon activation.

Accumulation of tRNA Fragments and Operon Induction Occur through at Least Two Pathways, One of Which Requires RecA

Many of the identified genes, such as *ruvA, polA, ruvC, yebC, uvrD* and *ftsK,* were also isolated in a screen for *E. coli* mutations that result in expression of the SOS regulon, a gene network induced upon DNA damage (O'Reilly and Kreuzer, 2004). To determine if the SOS response was required for tRNA fragment accumulation or activation of the *rsr-yrlBA-rtcBA* operon, we examined strains lacking RecA, since expression of the SOS regulon initiates when activated RecA assists cleavage of the LexA repressor. Notably, the tRNA$^{Trp(CCA)}$ fragments that accumulated

occurs in these cells due to lack of pseudouridine in some anticodon stems. Activation of the *rsr-yrlBA-rtcBA* operon in Δ*truA* strains was also unaffected by *recA* deletion (Figure 3E). Our results support a model in which tRNA fragments can be generated through at least two pathways, one involving tRNA fragility and a second requiring the RecA component of the SOS response. In this model, accumulation of tRNA fragments may result, directly or indirectly, in production of a ligand that binds the RtcR CARF domain to activate the operon. Consistent with this hypothesis, operon activation in both Δ*truA* and Δ*ruvA* cells requires RtcR (Figures 3F and 3G).

DNA Damaging Agents Cause tRNA Cleavage and Operon Activation

To obtain further evidence that tRNA fragment accumulation was linked to operon activation, we asked if treatment with DNA damaging agents results in tRNA cleavage. Upon treatment with the interstrand crosslinker mitomycin C (MMC), which leads to operon activation (Kurasz et al., 2018), FLAG$_3$-Rsr expression was detected within one hour and peaked by 2 h (Figure 4A). Cleavage of the LexA repressor occurred with similar kinetics.

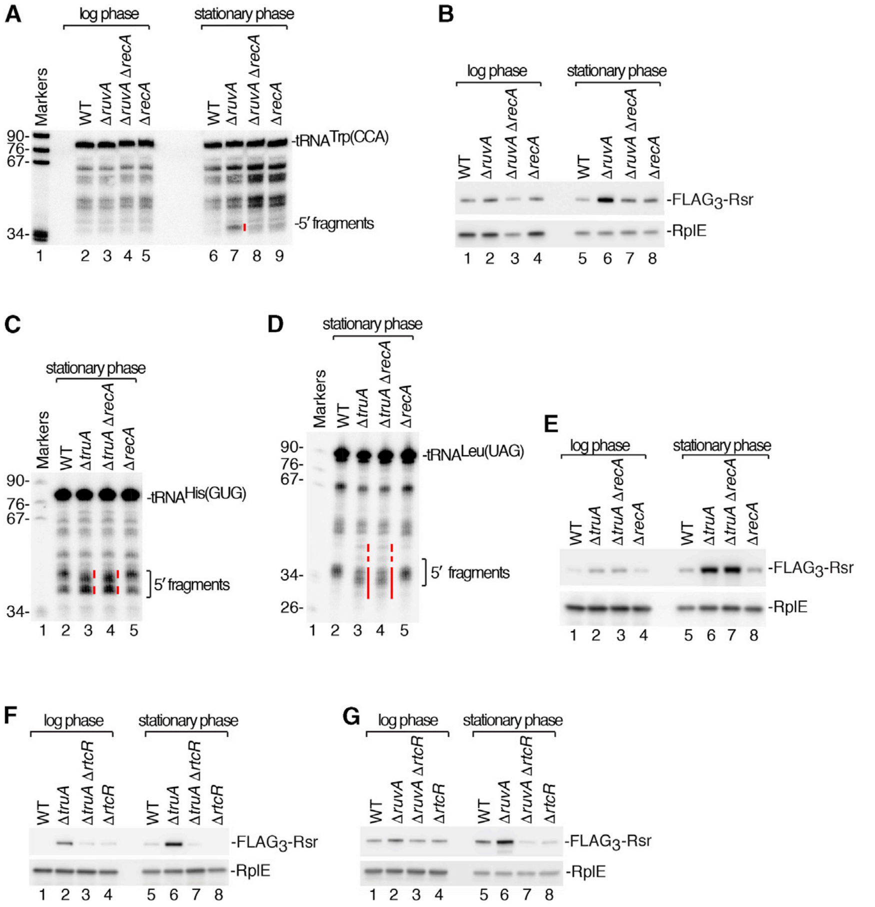

Figure 3. Accumulation of tRNA Fragments and Operon Activation Occur via Two Pathways

(A) RNA from strains grown to mid-log or stationary phase was subjected to northern blotting to detect $tRNA^{Trp(CCA)}$ 5′ halves. Lane 1, size markers (nt).

(B) Immunoblots were performed on lysates from the indicated *$FLAG_3$-rsr* strains to detect $FLAG_3$-Rsr. RplE, loading control.

(C and D). RNA from the indicated strains grown to stationary phase was subjected to northern blotting to detect 5′ halves of $tRNA^{His(GUG)}$ (C) and $tRNA^{Leu(UAG)}$ (D).

(E–G) Lysates from the indicated strains were immunoblotted to detect $FLAG_3$-Rsr. RplE, loading control.

In (A), (C), and (D), red lines denote fragments differing in mobility or levels in mutant strains.

Operon induction required RecA and LexA cleavage, as $FLAG_3$-Rsr was not detected in Δ*recA* strains or strains carrying the uncleavable *lexA3* allele (Little and Harper, 1979) (Figures 4A and S3A). (Deletions of *lexA* are lethal, because *S.* Typhimurium contains LexA-regulated prophages; Bunny et al., 2002; Lemire et al., 2011.) Similar to Δ*ruvA* and Δ*truA* strains, operon expression required RtcR (Figure S3B).

Upon MMC treatment, many tRNAs, including $tRNA^{Trp(CCA)}$, $tRNA^{Tyr(GUA)}$, and $tRNA^{Cys(GCA)}$, underwent cleavage, as both 5′ (Figures 4B–4H) and 3′ (Figures S3D and S3E) fragments accumulated. Fragments were detected within 1 h of adding MMC but were absent or reduced in Δ*recA* and *lexA3* strains (Figures 4B–4D and S3C–S3E). Levels of some full-length tRNAs, such as $tRNA^{Trp(CCA)}$, decreased concomitantly (Figures 4E and S3D), indicating the fragments derive from cleavage of mature tRNAs rather than reduced decay of preexisting fragments. Although the operon-encoded YrlA RNA contains a tRNA-like domain (Chen et al., 2014), fragments of this RNA were not detected (Figure S3F), indicating it is not a substrate for the RecA-dependent nuclease. Operon activation and tRNA fragment accumulation also occurred upon treatment with other DNA damaging agents, such as bleomycin, which cleaves single- and double-stranded DNA (Bolzán and Bianchi, 2018), and methyl methanesulfonate (MMS), which methylates DNA (Wyatt and Pittman, 2006) (Figures 4I, S3G, and S3H). Together, our data indicate that a tRNA anticodon nuclease is activated as part of the *S.* Typhimurium SOS response.

Inspection of the tRNA fragments that accumulated upon MMC treatment revealed some differences between the mutant strains. Most 5′ and 3′ fragments increased in cells lacking PNPase (Figures 4E–4G and S3E, lane 10). For some tRNAs that are *truA* substrates, the fragments that accumulated were altered in mobility in Δ*truA* strains compared to wild-type cells and increased further in Δ*truA* Δ*pnp* strains (Figure 4H, lanes 9 and 14, red lines), implicating PNPase in their degradation. Fragments of $tRNA^{Trp(CCA)}$ and $tRNA^{Tyr(GUA)}$ were slightly increased in Δ*rtcB* Δ*pnp* strains compared to Δ*pnp* strains (Figures 4E, 4F, and S3D, lanes 10 and 15), supporting a possible role for RtcB in repairing broken tRNAs.

To further assess the link between tRNA fragments and operon activation, we examined the *E. coli rtcBA* operon, which is regulated by RtcR. Overexpression of two toxins that cleave tRNAs, but not MMC incubation, activates this operon (Engl et al., 2016; Kurasz et al., 2018). If tRNA fragments are important for activation, then failure of the *E. coli* operon to be expressed in the presence of MMC may be due to the absence of these fragments. Consistent with our model, tRNA fragments did not accumulate in *E. coli* during growth in MMC (Figures S3I and S3J). Western blotting to detect RtcB confirmed that the *E. coli* operon was not induced (Figure S3K).

Together, our results reveal that RecA-dependent tRNA cleavage and *rsr-yrlBA-rtcBA* operon activation occur during conditions that induce the SOS response in *S*. Typhimurium. Since tRNA fragments do not accumulate when the operon is activated by overexpressing constitutively active RtcRΔN (Figures 4E–4H, S3D, and S3E, lane 2), operon activation in itself does not cause fragment accumulation. Instead, this result, together with our data that the operon is activated in Δ*truA* strains in the absence of DNA damage, supports a model in which tRNA cleavage results in a ligand that activates the operon.

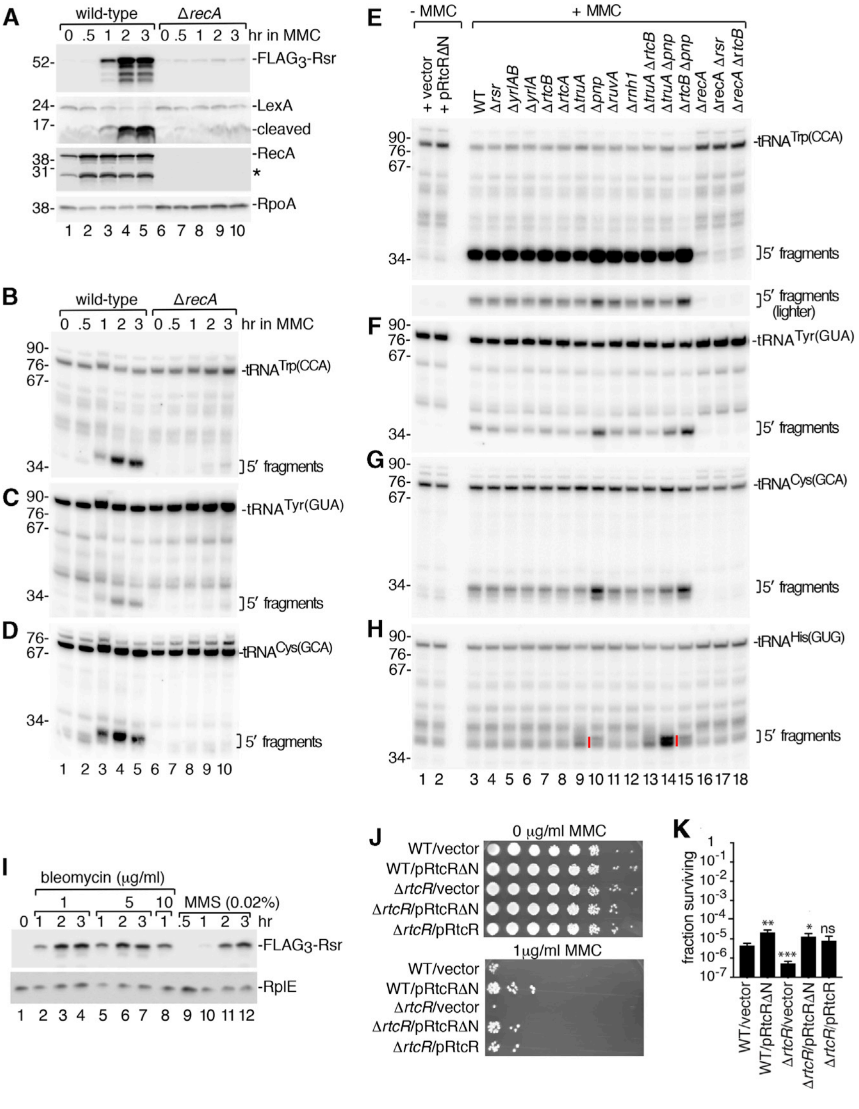

Figure 4. DNA Damaging Agents Result in tRNA Cleavage and Operon Activation

(A) After growing wild-type and Δ*recA FLAG$_3$-rsr* strains in MMC for the indicated times, FLAG$_3$-Rsr, LexA, RecA, and RpoA (loading control) were detected on immunoblots. Asterisk, RecA fragment.

(B–D) After growing wild-type and Δ*recA* strains in MMC, northern analysis was performed to detect 5′ halves of the indicated tRNAs.

(E–H) RNA from strains grown in MMC was subjected to northern analysis to detect 5′ halves of the indicated tRNAs (lanes 3–18). Lanes 1 and 2, RNA from wild-type cells carrying empty vector or pRtcRΔN, respectively. In (E), the bottom panel is a lighter view of the 5′ halves. In (H), red lines denote fragments differing in mobility in Δ*truA* and Δ*truA* Δ*pnp* strains.

(I) After treating *FLAG$_3$-rsr* strains with bleomycin or MMS for 0.5, 1, 2, or 3 h, lysates were subjected to immunoblotting to detect FLAG$_3$-Rsr and RplE.

(J) After growth of the indicated strains in 0 (top) and 1 μg/mL MMC (bottom panel) for 2 h, serial 10-fold dilutions were spotted on Luria-Bertani broth (LB) agar and grown at 37°C.

(K) Aliquots of the cells in (J) were plated on LB agar and colonies counted to determine the fraction of surviving cells. Data are mean (n = 3) ± SEM. p values were calculated with two-tailed unpaired t tests. *$p < 0.05$, **$p < 0.01$, and ***$p < 0.001$; ns, not significant.

See also Figure S3.

We determined whether expression of the *rsr-yrlBA-rtcBA* operon is important for survival after MMC exposure. Wild-type strains expressing constitutively active RtcR (RtcRΔN) were more resistant to 1 μg/mL MMC than the same cells carrying an empty vector, while strains lacking RtcR (Δ*rtcR*) were less resistant than wild-type cells (Figure 4J). Quantitation revealed that wild-type strains overexpressing RtcR were 4.8-fold more resistant than cells carrying an empty vector, while Δ*rtcR* cells were 9.4-fold less resistant than wild-type cells (Figure 4K). The decreased resistance of Δ*rtcR* strains could be complemented by expressing either RtcRΔN or wild-type RtcR on a plasmid. Thus, operon activation confers a growth advantage in MMC.

Most tRNA Cleavage Occurs in the Anticodon Loop, Leaving a Cyclic Phosphate End

To understand how tRNA cleavage could result in a ligand, we characterized the fragments that accumulate upon DNA damage. Since endonucleases can leave 2′, 3′-cyclic phosphate, 3′-phosphate, or 3′-OH at the 3′ end of the 5′ fragment, we tested if pre-treatment with T4 polynucleotide kinase (PNK), which converts 2′, 3′-cyclic phosphate and 3′-phosphate to 3′-OH, was needed for T4 RNA ligase (which requires 3′-OH) to ligate a 5′-phosphate-containing oligonucleotide to the 5′ fragments (Figure S4A). Comparison of RNA from wild-type and Δ*pnp* strains, followed by northern blotting to detect specific tRNAs, revealed that ligation to the 5′ fragments of all examined tRNAs increased in the presence of PNK (Figures S4B–S4E). To distinguish between 3′-phosphate and cyclic phosphate, we asked if treatment with calf intestinal phosphatase (CIP) (which removes 3′-phosphate, but not 2′, 3′-cyclic phosphate) could substitute for PNK or if treatment with acid (which opens up cyclic phosphate) was also required. Maximal ligation of the oligonucleotide to the 5′ tRNA fragments occurred in the presence of acid and CIP, indicating some fragments end in cyclic phosphate (Figures S4G–S4J). Thus, one or more metal-independent endoribonucleases (which leave 2′, 3′-cyclic phosphate) (Yang, 2011) contribute to cleavage. As some ligation occurred with CIP alone, 5′ fragments ending in 3′ phosphate were also present (Figures S4G–S4J). These species could derive from fragments ending in 2′, 3′-cyclic phosphate or from cleavage by additional nucleases.

Using 3′ rapid amplification of cDNA ends, we determined that cleavage occurred within the anticodon loops. For tRNA$^{Trp(CCA)}$, tRNA$^{Tyr(GUA)}$, tRNA$^{Cys(GCA)}$, and tRNA$^{Phe(GAA)}$, most 5′ fragments terminated at position 36, the last nucleotide of the anticodon,

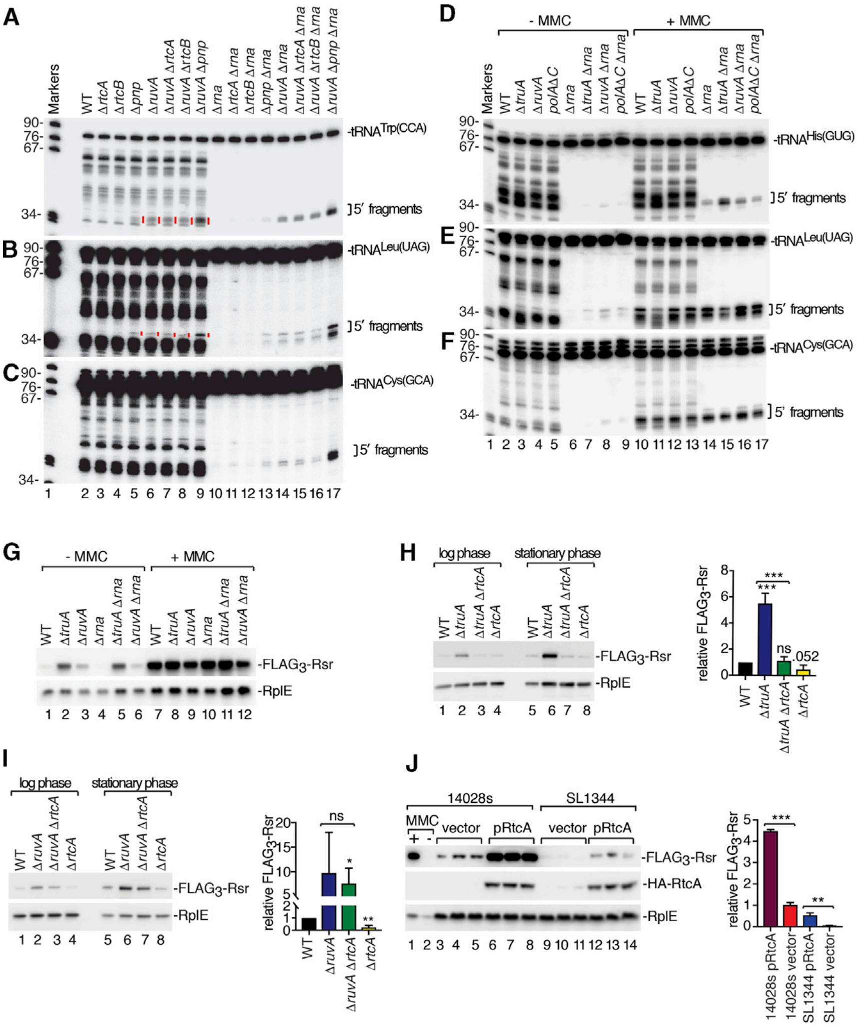

Figure 5. RtcA Is Important for Operon Activation

(A–C) RNA from the indicated strains was subjected to northern blotting to detect 5′ halves of tRNA$^{Trp(CCA)}$ (A), tRNA$^{Leu(UAG)}$ (B), and tRNA$^{Cys(GCA)}$ (C). In (A) and (B), specific fragments that become evident in Δ*rna* strains are denoted by red lines in strains containing RNase I (lanes 5–8).

(D–F) After growing the indicated strains without or with MMC, RNA was subjected to northern blotting to detect 5′ halves of tRNA$^{His(GUG)}$ (D), tRNA$^{Leu(UAG)}$ (E) and tRNA$^{Cys(GCA)}$ (F).

(G) After growing the indicated *FLAG$_3$-rsr* strains without or with MMC, lysates were subjected to immunoblotting to detect FLAG$_3$-Rsr and RplE.

(H and I) Lysates of *FLAG$_3$-rsr* strains carrying the indicated deletions were immunoblotted to detect FLAG$_3$-Rsr. RplE, loading control. Right, quantitation (n = 3).

(J). Lysates of 14028s and SL1344 *FLAG$_3$-rsr* strains carrying empty vector (lanes 3–5 and 9–11) or pHA-RtcA expressing RtcA fused to a hemagglutinin (HA) epitope tag (lane 6–8 and 12–14) were subjected to immunoblotting to detect FLAG$_3$-Rsr, HA-RtcA, and RplE. Right, quantitation (n = 3).

Data in (H), (I), and (J) are mean (n = 3) ± SEM. p values were calculated with two-tailed unpaired t tests. *$p < 0.05$, **$p < 0.01$, and ***$p < 0.001$; ns, not significant. See also Figures S4 and S5 and Table S2.

with the apparent cleavage site between two adenines (Figure S4K, arrowheads; Table S2). For tRNA^{fMet}, most cleavage occurred after position 37, between two adenines (Figure S4K; Table S2). For tRNA$^{Leu(UAG)}$, cleavage was heterogenous, with fragments ending at multiple sites (Figure S4K, arrows; Table S2). Slightly shorter 5′ fragments of most tRNAs were also recovered, which may represent exonucleolytic nibbling and/or other cleavages (arrows).

We tested if the abundant metal-independent endonuclease RNase I was important for cleavage. In strains lacking RNase I (Δ*rna*), we observed striking decreases in all nonspecific fragments (Figure 5A, lanes 10–17). However, the specific tRNA$^{Trp(CCA)}$ and tRNA$^{Leu(UAG)}$ fragments that accumulate in Δ*ruvA* strains were unchanged in Δ*ruvA* Δ*rna* strains (Figures 5A and 5B, lanes 6 and 14). Although we could not detect specific tRNA$^{Cys(GCA)}$ or tRNA$^{Tyr(GUA)}$ fragments in Δ*pnp* and Δ*ruvA* strains due to the background of nonspecific fragments, specific fragments were evident in Δ*pnp* Δ*rna* and Δ*ruvA* Δ*rna* strains (Figures 5C and S5A, lanes 13 and 14). The RecA-dependent tRNA fragments detected in MMC were also unaffected (Figures 5D–5F and S5B, lanes 14–17). As expected if these fragments are important for formation of the ligand that binds RtcR, operon induction was unaffected in Δ*rna* strains (Figure 5G).

Despite much effort, we were unable to identify the endonuclease responsible for the RecA-dependent tRNA cleavage. We examined strains lacking 22 other toxins and potential nucleases, including three metal-independent nucleases encoded adjacent to consensus LexA sites. These are YafQ, the toxin component of an antitoxin:-toxin cassette encoded downstream of the *rsr-yrlBA-rtcBA* operon (Figure S5C); HigB, which is encoded with its HigA antitoxin downstream of a LexA-regulated ORF; and HigB2, which is encoded on the opposite strand such that HigB and HigB2 may be controlled through the same palindromic LexA site (Figure S5D). All three toxins are part of the RelE family, which bind ribosomes and cleave translating mRNAs (Harms et al., 2018). Northern and western blotting of Δ*ruvA* strains carrying deletions of all three toxins revealed that they were not required for accumulation of tRNA$^{Trp(CCA)}$ fragments or operon activation (Figures S5E and S5F). We tested strains deleted for 19 other toxins and potential nucleases, with similar negative results (Figures S5G–S5J). Since RNA decay pathways are often redundant (Houseley and Tollervey, 2009), multiple endonucleases may carry out tRNA cleavage.

RtcA Is Important for Induction of the *rsr-yrlBA-rtcBA* Operon

To test if 2′, 3′-cyclic phosphate RNA ends were important for operon activation, we examined the role of RtcA, which converts

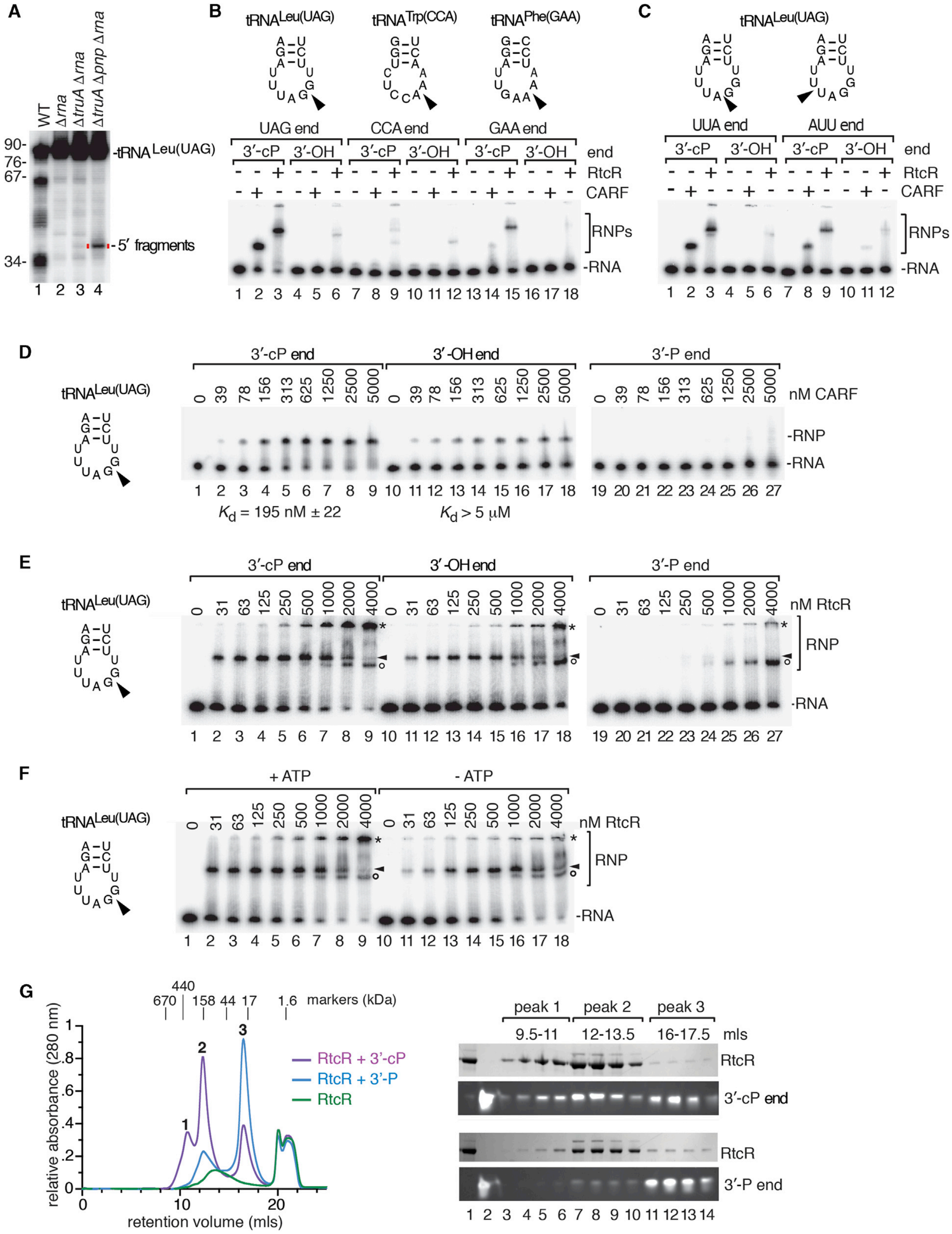

(legend on next page)

3′-phosphate ends to 2′, 3′-cyclic phosphate (Genschik et al., 1998). Operon activation decreased 4.8-fold in Δ*truA* Δ*rtcA* cells compared to Δ*truA* cells and was similar to wild-type cells (Figure 5H). Thus, the lower stability of some tRNA anticodon stems in Δ*truA* strains may make them susceptible to nucleases that leave 3′-phosphate ends that are subsequently converted by RtcA to 2′, 3′-cyclic phosphate. In contrast, operon activation was not significantly different between Δ*ruvA* and Δ*ruvA* Δ*rtcA* strains (Figure 5I), supporting the idea that tRNA halves that accumulate in Δ*ruvA* strains derive from cleavage by a metal-independent endonuclease.

We also found that the low level of expression of the *rsr-yrlBA-rtcBA* operon in wild-type 14028s strains in stationary phase was reduced in Δ*rtcA* strains (Figures 5H and 5I). This suggested that the RtcA levels in 14028s, but not SL1344 strains, might be sufficient to convert broken tRNA ends to 2′, 3′-cyclic phosphates as part of a feedback loop resulting in low levels of operon expression. To test if increased RtcA levels were sufficient for operon activation, we expressed RtcA under control of the arabinose-inducible promoter in the SL1344 and 14028s strains. We detected RtcA in both strains in the absence of arabinose due to the leakiness of this promoter (Figure 5J). Importantly, operon expression increased, as Rsr levels were 4.5-fold higher in 14028s strains and 11-fold higher in SL1344 strains (Figure 5J) compared to the same cells carrying empty vector. We conclude that RtcA expression is sufficient to activate the *rsr-yrlBA-rtcBA* operon. As expression of the operon in the SL1344 strain did not reach that of 14028s, additional differences between these strains contribute to operon activation.

The RtcR CARF Domain Binds tRNA Fragments with Cyclic Phosphate Ends

A model that accommodates all our data is that 5′ tRNA fragments ending in 2′, 3′-cyclic phosphate are the ligands that bind the CARF domain of RtcR to activate operon transcription. However, since many tRNAs undergo cleavage during growth in MMC, it was unclear which 5′ fragments could be responsible. To reduce the candidates, we used northern blotting to identify the 5′ tRNA fragments that accumulate in Δ*truA* strains, since only some tRNAs are TruA substrates. To assist visualization, we analyzed strains that also lacked RNase I. Of the tRNAs assayed (tRNA$^{Gln(CUG)}$, tRNA$^{His(GUG)}$, tRNA$^{Leu(UAG)}$, tRNA$^{Leu(UAA)}$, tRNA$^{Leu(CAA)}$, tRNA$^{Cys(GCA)}$, tRNA$^{Tyr(GUA)}$, and tRNA$^{Phe(GAA)}$), the most prominent fragments were from tRNA$^{Leu(UAG)}$ (Figure 5E, lane 7). These fragments increased when PNPase was also deleted (Figure 6A), consistent with the enhanced operon activation in Δ*truA* Δ*pnp* strains (Figure 2A). The fragments ended in cyclic phosphate, as ligation of an oligonucleotide to most tRNA$^{Leu(UAG)}$ 5′ fragments required treatment with acid and CIP (Figure S6A). Thus, tRNA$^{Leu(UAG)}$ 5′ fragments were good candidates for a ligand that activates the operon.

We tested whether 5′ fragments of tRNA$^{Leu(UAG)}$ or other tRNAs could bind the CARF domain. We expressed both the CARF domain and RtcR in *E. coli* (Figures S6B and S6C) and performed electrophoretic mobility shift assays (EMSAs) with the purified proteins. Initial studies in which we incubated 5′ tRNA halves derived from tRNA$^{Leu(UAG)}$, tRNA$^{Trp(CCA)}$, and tRNA$^{Phe(GAA)}$ ending in 2′, 3′-cyclic phosphate or 3′-OH revealed that both the isolated CARF domain and RtcR bound most efficiently to 5′ halves of tRNA$^{Leu(UAG)}$ (Figure 6B). We also tested tRNA$^{Leu(UAG)}$ 5′ halves ending at other nucleotides in the anticodon loop. Although all tested 5′ halves ending in cyclic phosphate bound both the CARF domain and RtcR, fragments that ended after the G of the UAG were most efficiently bound (Figures 6B and 6C). Quantitative assays revealed that the amount of CARF domain required to shift 50% of these tRNA$^{Leu(UAG)}$ halves ending in cyclic phosphate was ~200 nM, while more than 5 μM was required to shift 50% of the same RNAs ending in in 3′-OH (Figure 6D). Specific binding to the CARF domain was not detected when the fragments ended in 3′-phosphate.

We also examined binding to full-length RtcR, in which the CARF domain is followed by AAA+ (ATPases associated with multiple cellular activities) and helix-turn-helix DNA-binding domains. Based on other bacterial enhancer proteins, RtcR is expected to be a dimer in its unliganded form. In the absence of ligand, the CARF domain negatively regulates transcription, most likely by preventing the AAA+ domain from oligomerizing (Genschik et al., 1998; Bush and Dixon, 2012). The amount of RtcR required to shift 50% of the tRNA$^{Leu(UAG)}$ fragments ending in cyclic phosphate was slightly less than that of the isolated CARF domain (~125 nM; Figure 6E, arrowheads). Beginning at 125 nM RtcR, we also detected a larger complex. This complex, which could represent oligomerization, eventually became the predominant species (Figure 6E, asterisk). Formation of both

Figure 6. RtcR Oligomerizes on Binding tRNA Fragments Ending in Cyclic Phosphate

(A) RNA from the indicated strains was subjected to northern blotting to detect tRNA$^{Leu(UAG)}$ 5′ halves.

(B) ^{32}P-labeled 5′ tRNA halves (1 nM) ending after the anticodon in 2′, 3′-cyclic phosphate (lanes 1–3, 7–9, and 13–15) or 3′-OH (lanes 4–6, 10–12, and 16–18) were incubated with no protein, 0.5 μM CARF domain, or 0.5 μM RtcR. Reactions contained 1 mM ATP. RNA-protein complexes (RNPs) were separated from naked RNA in native gels.

(C) Similar to (B), except that 5′ tRNA$^{Leu(UAG)}$ halves ended at the indicated positions.

(D and E) 5′ tRNA$^{Leu(UAG)}$ halves ending after the G of the anticodon were incubated with the indicated concentrations of CARF domain (D) or RtcR (E). RNAs ended in 2′, 3′-cyclic phosphate (lanes 1–9), 3′-OH (lines 10–18), or 3′-phosphate (lanes 19–27). In (E), arrowheads denote the first complexes formed, while asterisks denote complexes that could represent oligomers. Circles, complexes that form on all three RNAs at the highest RtcR concentrations. In (D) and (E), the samples were fractionated in two gels. Binding reactions in (E) contained 1 mM ATP.

(F) tRNA$^{Leu(UAG)}$ halves ending in cyclic phosphate were mixed with the indicated concentrations of RtcR with or without 1 mM ATP. Complexes are designated with arrowheads, asterisks, and circles as in (E).

(G) Size exclusion chromatography was performed on RtcR (48 μM) alone or bound to 48 μM 5′ tRNA$^{Leu(UAG)}$ halves ending in 3′-phosphate or 2′, 3′-cyclic phosphate. Left, overlay of chromatograms. Right, proteins and RNA in the indicated peaks were fractionated in SDS-PAGE and denaturing polyacrylamide gels, respectively. The complex eluting at 20 mL is ATP from the binding reaction.

See also Figure S6.

complexes, but not the complex formed with the isolated CARF domain, was enhanced when ATP was present, suggesting conformational changes mediated by the AAA+ domain contribute to their formation (Figures 6F and S6D). As both RtcR-containing complexes were reduced in levels when the fragments ended in 3′-OH and were barely detectable when the fragments ended in 3′-phosphate, tRNA 5′ halves ending in cyclic phosphate are the preferred ligand for their formation. At the highest protein concentrations, a third complex that migrated slightly faster than the initial complex was detected (Figure 6E, circles). As formation of this complex was not dependent on the presence of either ATP or a cyclic phosphate RNA end, this complex may represent nonspecific interactions of the RNA fragment with RtcR.

To further characterize these complexes, we performed size exclusion chromatography and analyzed the composition of the peaks using gel electrophoresis. In these experiments, we mixed RtcR with equimolar amounts of 5′ $tRNA^{Leu(UAG)}$ halves to maximize complex formation. When the tRNA halves ended in cyclic phosphate, two RtcR/tRNA complexes formed that migrated at ~400 and 160 kDa, consistent with hexameric (358 kDa) and dimeric (119 kDa) forms of RtcR bound to three and one tRNA halves, respectively (12 kDa each). A third peak consisted of unbound tRNA halves (Figure 6G). Notably, when the tRNA halves terminated in 3′-phosphate, the 400-kDa complex was not detected, the 160-kDa complex was reduced, and the peak corresponding to unbound RNA increased (Figure 6G). We conclude that RtcR forms oligomers upon binding tRNA fragments ending in cyclic phosphate.

DISCUSSION

Although *rtcBA* and *rsr-yrlBA-rtcBA* RNA repair operons are widespread in bacteria, the signals that activate the operons have been obscure. We showed that tRNA 5′ fragments ending in 2′, 3′-cyclic phosphate bind the RtcR CARF domain. These tRNA fragments accumulate in the presence of mutations in genes that maintain tRNA homeostasis and upon activation of a LexA-regulated endoribonuclease. Since operon expression is important for *S*. Typhimurium survival after MMC exposure, our experiments uncover a signaling pathway involving tRNA and implicate *rsr-yrlBA-rtcBA* operon components in the repair of nucleic acid damage.

A Signaling Pathway Involving tRNA

Our data support a model in which 5′ tRNA fragments ending in 2′, 3′-cyclic phosphate bind the RtcR CARF domain, triggering oligomerization of the AAA+ domain. These fragments accumulate in Δ*truA* strains, which contain hypomodified tRNAs with unstable anticodon arms (Figure 7A), in strains carrying mutations that result in DNA damage and in wild-type cells treated with DNA damaging agents (Figure 7B). These tRNA fragments also accumulate in strains lacking PNPase (Figure 7C). Binding of the fragments to RtcR triggers oligomerization, resulting in operon transcription (Figure 7D). Our proposal that 5′ tRNA fragments ending in 2′, 3′-cyclic phosphate are critical ligands is consistent with data that the *E. coli* operon is activated by ectopic expression of colicin D and *S*. Typhimurium LT2 VapC (Engl et al., 2016), toxins that cleave the anticodon loops of $tRNA^{Arg}$ and $tRNA^{Met}$, respectively (Masaki and Ogawa, 2002; Winther and Gerdes, 2011).

Although canonical CARF domains, such as those of Csm6 and Csx1, dimerize to form a symmetric binding pocket for cOA (Jia et al., 2019; Molina et al., 2019; Garcia-Doval et al., 2020), structural analyses will be required to determine how the divergent RtcR CARF domain recognizes asymmetric tRNA fragments ending in cyclic phosphate. As the affinity of the RtcR CARF domain for the *in-vitro*-synthesized tRNA halves used in our assays is lower than the 0.5–5 nM affinity of canonical CARF domains for cOA ligands (Kazlauskiene et al., 2017; Niewoehner et al., 2017), other proteins and/or tRNA modifications, which are often present in the vicinity of the anticodon, may contribute to recognition. It is also possible that another tRNA 5′ half may be a better ligand than the $tRNA^{Leu(UAG)}$ fragment used in our studies or that tRNA 3′ fragments contribute.

If binding of 5′ tRNA fragments ending in 2′, 3′-cyclic phosphate activates the operon, how is signaling regulated? For CARF domains activated by cOA, signaling is terminated by ring nuclease activity that is encoded within the CARF domain (Athukoralage et al., 2019; Jia et al., 2019) or supplied by standalone nucleases (Athukoralage et al., 2018). One possibility is that RtcA and RtcB function as part of a feedback loop to ligate cleaved tRNAs and terminate operon expression. PNPase may contribute to reducing operon expression by degrading tRNA fragments (Figure 7C). However, since RNAs ending in 3′-phosphate or 2′, 3′-cyclic phosphate are not PNPase substrates (Singer, 1958), other enzymes must first convert these ends to 3′-OH.

It is curious that tRNA cleavage occurs upon DNA damage in *S*. Typhimurium. Since *S*. Typhimurium contains LexA-regulated prophages (Lemire et al., 2011), prophage-encoded endonucleases may carry out cleavage. In this case, the decreased levels of specific tRNAs may be peripheral to the DNA damage response, instead allowing the phage to reduce host protein synthesis or favor translation of specific proteins. It is also possible that tRNA cleavage, by decreasing protein synthesis and slowing division, allows more time for DNA repair. Given the growing evidence that RNA can template and otherwise enhance DNA repair (Michelini et al., 2018), another possibility is that the tRNA halves play a role in restoring genome integrity.

Functions of the *rsr-yrlBA-rtcBA* Operon

Our findings that (1) the operon is activated by tRNA cleavage, (2) RtcA is required for operon activation in Δ*truA* cells, and (3) some tRNA fragments are present at slightly higher levels in Δ*rtcB* Δ*pnp* cells than Δ*pnp* cells support a role for RtcA and RtcB in tRNA repair (Figure 7E). Such a role would be consistent with the finding that RtcB seals anticodon loops after excision of intervening sequences in eukaryotes and archaea (Englert et al., 2011; Popow et al., 2011; Kosmaczewski et al., 2014). It would also be consistent with our observation that mutations in RtcB trigger operon expression, since tRNA halves might be expected to accumulate in Δ*rtcB* cells. Although we did not detect accumulation of tRNA halves in Δ*rtcB* cells, we did not examine the full complement of tRNAs. Our identification of

CellPress
OPEN ACCESS

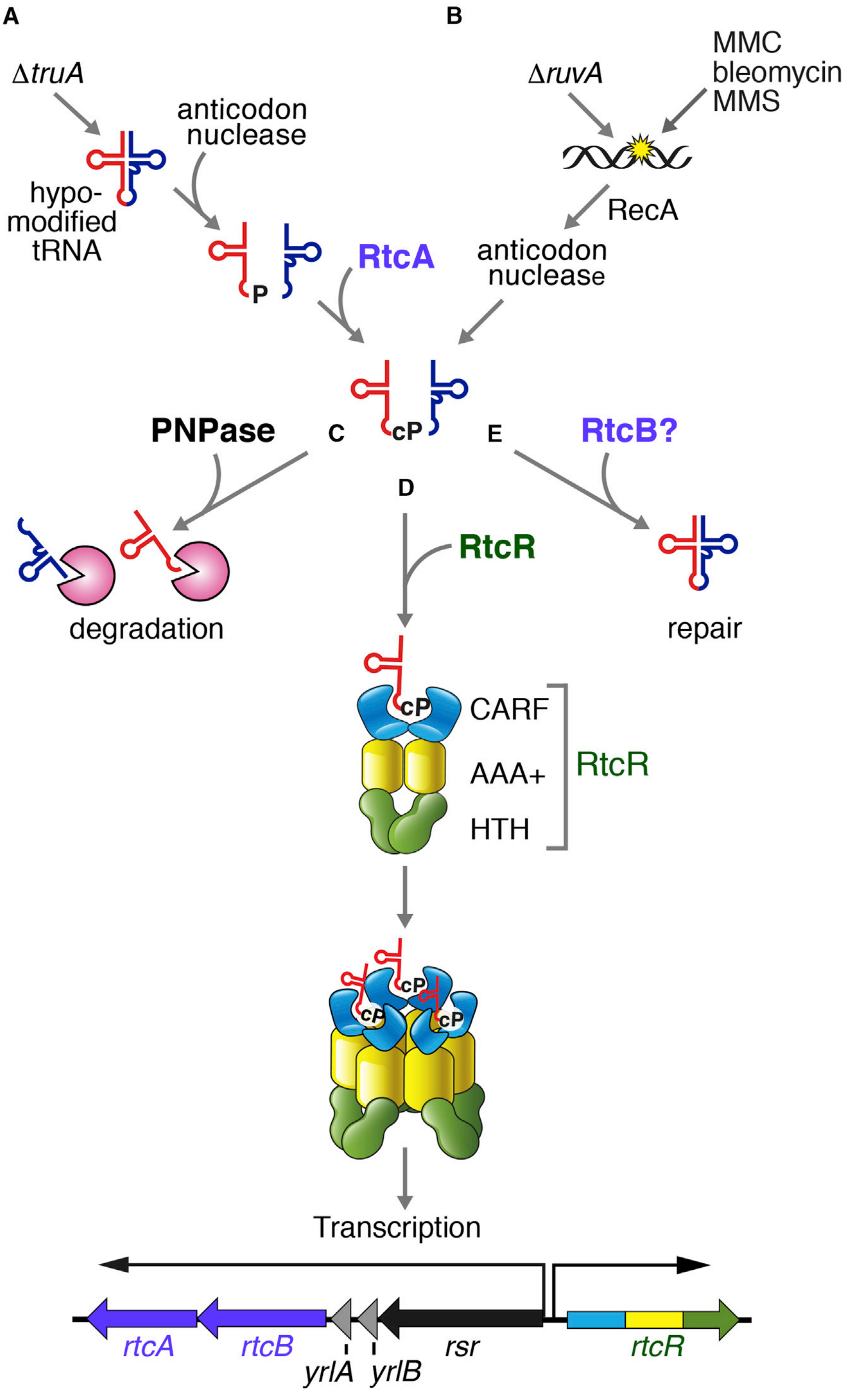

Figure 7. Model for Operon Activation

(A) In Δ*truA* strains, hypomodified tRNAs are susceptible to nuclease, leading to tRNA fragment accumulation. RtcA converts the ends of the 5′ fragments to 2′, 3′-cyclic phosphate (cP).

(B) In Δ*ruvA* strains and after treatment with DNA damaging agents, a RecA-regulated endonuclease cleaves tRNAs, resulting in 5′ halves ending in cyclic phosphate.

(C–E) We propose that 5′ tRNA halves ending in cyclic phosphate can be degraded by PNPase (C); bind the CARF domain of a RtcR dimer, resulting in oligomerization and operon activation (D); and may also be repaired by RtcB (E).

conditions that activate the operon should allow us to determine the diversity of RtcA and RtcB substrates.

Although our studies did not address the roles of Rsr and Y RNAs, several possibilities can be envisioned. Since *D. radiodurans* Rsr and Y RNA assist PNPase in degrading structured RNA (Chen et al., 2013), they could potentially function in tRNA decay. An alternative, but not exclusive, possibility is that the tRNA-like domain of YrlA RNA, which is not a substrate for the anticodon nuclease (Figure S3F), acts as a competitive inhibitor of this nuclease.

Finally, we note that RtcB, Rsr, and Y RNAs could potentially function in DNA repair. *E. coli* RtcB can ligate DNA 3′-PO_4 ends to DNA 5′-OH ends and can add a ppG cap to DNAs ending in 3′-phosphate, a modification that allows priming by DNA polymerase (Das et al., 2013, 2014). *D. radiodurans* Rsr contributes to survival after UV irradiation and Rsr and Y RNAs are among

the most upregulated genes during recovery of *D. radiodurans* from DNA damage (Chen et al., 2000; Tanaka et al., 2004). Since Rsr and Y RNAs are encoded adjacent to RtcB in diverse bacteria, it will be interesting to determine if activation in response to DNA damage is a conserved feature of *rsr-yrlBA-rtcBA* operons.

STAR★METHODS

Detailed methods are provided in the online version of this paper and include the following:

- KEY RESOURCES TABLE
- RESOURCE AVAILABILITY
  - Lead Contact
  - Materials Availability
  - Data and Code Availability
- EXPERIMENTAL MODEL AND SUBJECT DETAILS
- METHOD DETAILS
  - Transposon mutagenesis
  - β-Galactosidase assays
  - Construction of expression plasmids
  - Immunoblotting
  - RNA analyses and Northern blotting
  - RNA sequencing and analysis
  - Genome screening for candidate tRNA endoribonucleases
  - Survival assays
  - MazEF purification and MazF activation
  - Generation of 5′ tRNA halves for binding studies
  - Purification of full-length RtcR and the CARF domain
  - RNA binding assays
  - Size exclusion chromatography
- QUANTIFICATION AND STATISTICAL ANALYSIS
  - Statistical analysis

SUPPLEMENTAL INFORMATION

Supplemental Information can be found online at https://doi.org/10.1016/j.celrep.2020.108527.

ACKNOWLEDGMENTS

We thank Casey Fowler and Jorge Galan for the SL1344 strain, the pSRS_CM1/pSB4807 plasmid, and advice on the screen; Lynn Thomason and Donald Court for *E. coli* strains and advice; Eduardo Groisman, Issay Narumi, Isabella Moll, and Janice Zengel for other strains and antibodies; Eugene Koonin and Kira Makarova for help with identifying endonucleases; and Stephanie Shames, Eugene Valkov, Soyeong Sim, and Marco Boccitto for other helpful advice. We also thank Donald Court, Susan Gottesman, Traci Hall, Gisela Storz, Lynn Thomason, and members of the Wolin laboratory for comments on the manuscript. This work was supported in part by a National Science Foundation Graduate Research Fellowship (K.J.H.); National Institutes of Health grant R01 GM073863 (S.L.W.) and training grant T32 GM007223 (K.J.H.); and the Intramural Research Program, Center for Cancer Research, National Cancer Institute, National Institutes of Health (K.J.H., X.C., and S.L.W.).

AUTHOR CONTRIBUTIONS

K.J.H., X.C., and S.L.W. conceived the project and designed experiments. K.J.H. and X.C. performed the experiments, and K.J.H., X.C., and S.L.W. analyzed the resulting data. A.M.B. and L.A. performed bioinformatic analyses to identify potential endonucleases. The manuscript was written by K.J.H., X.C., and S.L.W.

DECLARATION OF INTERESTS

The authors declare no competing interests.

Received: July 20, 2020
Revised: November 3, 2020
Accepted: November 23, 2020
Published: December 22, 2020

SUPPORTING CITATIONS

The following references appear in the supplemental information: Blakely et al. (1993); Padan et al. (2005); Shiomi et al. (2008).

REFERENCES

Altschul, S.F., and Koonin, E.V. (1998). Iterated profile searches with PSI-BLAST: a tool for discovery in protein databases. Trends Biochem. Sci. *23*, 444–447.

Amitsur, M., Levitz, R., and Kaufmann, G. (1987). Bacteriophage T4 anticodon nuclease, polynucleotide kinase and RNA ligase reprocess the host lysine tRNA. EMBO J. *6*, 2499–2503.

Anantharaman, V., Makarova, K.S., Burroughs, A.M., Koonin, E.V., and Aravind, L. (2013). Comprehensive analysis of the HEPN superfamily: identification of novel roles in intra-genomic conflicts, defense, pathogenesis and RNA processing. Biol. Direct *8*, 15.

Aravind, L. (1999). An evolutionary classification of the metallo-beta-lactamase fold proteins. In Silico Biol. *1*, 69–91.

Arnez, J.G., and Steitz, T.A. (1994). Crystal structure of unmodified tRNA(Gln) complexed with glutaminyl-tRNA synthetase and ATP suggests a possible role for pseudo-uridines in stabilization of RNA structure. Biochemistry *33*, 7560–7567.

Athukoralage, J.S., Rouillon, C., Graham, S., Grüschow, S., and White, M.F. (2018). Ring nucleases deactivate type III CRISPR ribonucleases by degrading cyclic oligoadenylate. Nature *562*, 277–280.

Athukoralage, J.S., Graham, S., Grüschow, S., Rouillon, C., and White, M.F. (2019). A type III CRISPR ancillary ribonuclease degrades its cyclic oligoadenylate activator. J. Mol. Biol. *431*, 2894–2899.

Blakely, G., May, G., McCulloch, R., Arciszewska, L.K., Burke, M., Lovett, S.T., and Sherratt, D.J. (1993). Two related recombinases are required for site-specific recombination at dif and cer in E. coli K12. Cell *75*, 351–361.

Bolger, A.M., Lohse, M., and Usadel, B. (2014). Trimmomatic: a flexible trimmer for Illumina sequence data. Bioinformatics *30*, 2114–2120.

Bolzán, A.D., and Bianchi, M.S. (2018). DNA and chromosome damage induced by bleomycin in mammalian cells: An update. Mutat. Res. *775*, 51–62.

Bunny, K., Liu, J., and Roth, J. (2002). Phenotypes of lexA mutations in Salmonella enterica: evidence for a lethal lexA null phenotype due to the Fels-2 prophage. J. Bacteriol. *184*, 6235–6249.

Burroughs, A.M., and Aravind, L. (2016). RNA damage in biological conflicts and the diversity of responding RNA repair systems. Nucleic Acids Res. *44*, 8525–8555.

Bush, M., and Dixon, R. (2012). The role of bacterial enhancer binding proteins as specialized activators of σ54-dependent transcription. Microbiol. Mol. Biol. Rev. *76*, 497–529.

Byrne, R.T., Chen, S.H., Wood, E.A., Cabot, E.L., and Cox, M.M. (2014). *Escherichia coli* genes and pathways involved in surviving extreme exposure to ionizing radiation. J. Bacteriol. *196*, 3534–3545.

Callaghan, A.J., Marcaida, M.J., Stead, J.A., McDowall, K.J., Scott, W.G., and Luisi, B.F. (2005). Structure of Escherichia coli RNase E catalytic domain and implications for RNA turnover. Nature *437*, 1187–1191.

Chen, X., Quinn, A.M., and Wolin, S.L. (2000). Ro ribonucleoproteins contribute to the resistance of Deinococcus radiodurans to ultraviolet irradiation. Genes Dev. *14*, 777–782.

Chen, X., Wurtmann, E.J., Van Batavia, J., Zybailov, B., Washburn, M.P., and Wolin, S.L. (2007). An ortholog of the Ro autoantigen functions in 23S rRNA maturation in *D. radiodurans*. Genes Dev. *21*, 1328–1339.

Chen, X., Taylor, D.W., Fowler, C.C., Galan, J.E., Wang, H.W., and Wolin, S.L. (2013). An RNA degradation machine sculpted by Ro autoantigen and noncoding RNA. Cell *153*, 166–177.

Chen, X., Sim, S., Wurtmann, E.J., Feke, A., and Wolin, S.L. (2014). Bacterial noncoding Y RNAs are widespread and mimic tRNAs. RNA *20*, 1715–1724.

Cortese, R., Kammen, H.O., Spengler, S.J., and Ames, B.N. (1974). Biosynthesis of pseudouridine in transfer ribonucleic acid. J. Biol. Chem. *249*, 1103–1108.

Das, U., and Shuman, S. (2013). 2′-Phosphate cyclase activity of RtcA: a potential rationale for the operon organization of RtcA with an RNA repair ligase RtcB in *Escherichia coli* and other bacterial taxa. RNA *19*, 1355–1362.

Das, U., Chakravarty, A.K., Remus, B.S., and Shuman, S. (2013). Rewriting the rules for end joining via enzymatic splicing of DNA 3′-PO4 and 5′-OH ends. Proc. Natl. Acad. Sci. USA *110*, 20437–20442.

Das, U., Chauleau, M., Ordonez, H., and Shuman, S. (2014). Impact of DNA3′pp5′G capping on repair reactions at DNA 3′ ends. Proc. Natl. Acad. Sci. USA *111*, 11317–11322.

Davis, R.W., Botstein, D., and Roth, J.R. (1980). Advanced Bacterial Genetics: A Manual for Genetic Engineering (Cold Spring Harbor Laboratory Press).

Demarre, G., Guérout, A.M., Matsumoto-Mashimo, C., Rowe-Magnus, D.A., Marlière, P., and Mazel, D. (2005). A new family of mobilizable suicide plasmids based on broad host range R388 plasmid (IncW) and RP4 plasmid (IncPalpha) conjugative machineries and their cognate *Escherichia coli* host strains. Res. Microbiol. *156*, 245–255.

Dillingham, M.S., and Kowalczykowski, S.C. (2008). RecBCD enzyme and the repair of double-stranded DNA breaks. Microbiol. Mol. Biol. Rev. *72*, 642–671.

El-Gebali, S., Mistry, J., Bateman, A., Eddy, S.R., Luciani, A., Potter, S.C., Qureshi, M., Richardson, L.J., Salazar, G.A., Smart, A., et al. (2019). The Pfam protein families database in 2019. Nucleic Acids Res. *47* (*D1*), D427–D432.

Engl, C., Schaefer, J., Kotta-Loizou, I., and Buck, M. (2016). Cellular and molecular phenotypes depending upon the RNA repair system RtcAB of *Escherichia coli*. Nucleic Acids Res. *44*, 9933–9941.

Englert, M., Sheppard, K., Aslanian, A., Yates, J.R., 3rd, and Söll, D. (2011). Archaeal 3′-phosphate RNA splicing ligase characterization identifies the missing component in tRNA maturation. Proc. Natl. Acad. Sci. USA *108*, 1290–1295.

Fontaine, F., Gasiorowski, E., Gracia, C., Ballouche, M., Caillet, J., Marchais, A., and Hajnsdorf, E. (2016). The small RNA SraG participates in PNPase homeostasis. RNA *22*, 1560–1573.

Fowler, C.C., and Galan, J.E. (2018). Decoding a Salmonella Typhi regulatory network that controls typhoid toxin expression within human cells. Cell Host Microbe *23*, 65–76.e66.

Garcia-Doval, C., Schwede, F., Berk, C., Rostøl, J.T., Niewoehner, O., Tejero, O., Hall, J., Marraffini, L.A., and Jinek, M. (2020). Activation and self-inactivation mechanisms of the cyclic oligoadenylate-dependent CRISPR ribonuclease Csm6. Nat. Commun. *11*, 1596.

Genschik, P., Drabikowski, K., and Filipowicz, W. (1998). Characterization of the *Escherichia coli* RNA 3′-terminal phosphate cyclase and its sigma54-regulated operon. J. Biol. Chem. *273*, 25516–25526.

Gerdes, K., Howard, M., and Szardenings, F. (2010). Pushing and pulling in prokaryotic DNA segregation. Cell *141*, 927–942.

Goodman, A.L., McNulty, N.P., Zhao, Y., Leip, D., Mitra, R.D., Lozupone, C.A., Knight, R., and Gordon, J.I. (2009). Identifying genetic determinants needed to establish a human gut symbiont in its habitat. Cell Host Microbe *6*, 279–289.

Guzman, L.M., Belin, D., Carson, M.J., and Beckwith, J. (1995). Tight regulation, modulation, and high-level expression by vectors containing the arabinose PBAD promoter. J. Bacteriol. *177*, 4121–4130.

Harms, A., Brodersen, D.E., Mitarai, N., and Gerdes, K. (2018). Toxins, targets, and triggers: an overview of toxin-antitoxin biology. Mol. Cell *70*, 768–784.

Hoiseth, S.K., and Stocker, B.A. (1981). Aromatic-dependent *Salmonella typhimurium* are non-virulent and effective as live vaccines. Nature *291*, 238–239.

Hollis, T., and Shaban, N.M. (2011). Structure and function of RNase H enzymes. Nucleic Acids Mol. Biol. *26*, 299–317.

Houseley, J., and Tollervey, D. (2009). The many pathways of RNA degradation. Cell *136*, 763–776.

Iyer, L.M., Burroughs, A.M., Anand, S., de Souza, R.F., and Aravind, L. (2017). Polyvalent proteins, a pervasive theme in the intergenomic biological conflicts of bacteriophages and conjugative elements. J. Bacteriol. *199*, e00245-17.

Jarvik, T., Smillie, C., Groisman, E.A., and Ochman, H. (2010). Short-term signatures of evolutionary change in the *Salmonella enterica* serovar Typhimurium 14028 genome. J. Bacteriol. *192*, 560–567.

Jia, N., Jones, R., Yang, G., Ouerfelli, O., and Patel, D.J. (2019). CRISPR-Cas III-A Csm6 CARF domain is a ring nuclease triggering stepwise cA_4 cleavage with ApA > p formation terminating RNase activity. Mol. Cell *75*, 944–956.e6.

Johnson, L.S., Eddy, S.R., and Portugaly, E. (2010). Hidden Markov model speed heuristic and iterative HMM search procedure. BMC Bioinformatics *11*, 431.

Jurkin, J., Henkel, T., Nielsen, A.F., Minnich, M., Popow, J., Kaufmann, T., Heindl, K., Hoffmann, T., Busslinger, M., and Martinez, J. (2014). The mammalian tRNA ligase complex mediates splicing of XBP1 mRNA and controls antibody secretion in plasma cells. EMBO J. *33*, 2922–2936.

Kaimer, C., and Graumann, P.L. (2011). Players between the worlds: multifunctional DNA translocases. Curr. Opin. Microbiol. *14*, 719–725.

Kaniga, K., Bossio, J.C., and Galán, J.E. (1994). The *Salmonella typhimurium* invasion genes *invF* and *invG* encode homologues of the AraC and PulD family of proteins. Mol. Microbiol. *13*, 555–568.

Kawano, M., Aravind, L., and Storz, G. (2007). An antisense RNA controls synthesis of an SOS-induced toxin evolved from an antitoxin. Mol. Microbiol. *64*, 738–754.

Kazlauskiene, M., Kostiuk, G., Venclovas, Č., Tamulaitis, G., and Siksnys, V. (2017). A cyclic oligonucleotide signaling pathway in type III CRISPR-Cas systems. Science *357*, 605–609.

Kisker, C., Kuper, J., and Van Houten, B. (2013). Prokaryotic nucleotide excision repair. Cold Spring Harb. Perspect. Biol. *5*, a012591.

Kosmaczewski, S.G., Edwards, T.J., Han, S.M., Eckwahl, M.J., Meyer, B.I., Peach, S., Hesselberth, J.R., Wolin, S.L., and Hammarlund, M. (2014). The RtcB RNA ligase is an essential component of the metazoan unfolded protein response. EMBO Rep. *15*, 1278–1285.

Kurasz, J.E., Hartman, C.E., Samuels, D.J., Mohanty, B.K., Deleveaux, A., Mrázek, J., and Karls, A.C. (2018). Genotoxic, metabolic, and oxidative stresses regulate the RNA repair operon of *Salmonella enterica* serovar Typhimurium. J. Bacteriol. *200*, e00476–e00418.

Langmead, B., and Salzberg, S.L. (2012). Fast gapped-read alignment with Bowtie 2. Nat. Methods *9*, 357–359.

Lassmann, T. (2019). Kalign 3: multiple sequence alignment of large data sets. Bioinformatics *36*, 1928–1949.

Lemire, S., Figueroa-Bossi, N., and Bossi, L. (2011). Bacteriophage crosstalk: coordination of prophage induction by trans-acting antirepressors. PLoS Genet. *7*, e1002149.

Li, Z., Reimers, S., Pandit, S., and Deutscher, M.P. (2002). RNA quality control: degradation of defective transfer RNA. EMBO J. *21*, 1132–1138.

Little, J.W., and Harper, J.E. (1979). Identification of the *lexA* gene product of *Escherichia coli* K-12. Proc. Natl. Acad. Sci. USA *76*, 6147–6151.

Majorek, K.A., Dunin-Horkawicz, S., Steczkiewicz, K., Muszewska, A., Nowotny, M., Ginalski, K., and Bujnicki, J.M. (2014). The RNase H-like superfamily:

new members, comparative structural analysis and evolutionary classification. Nucleic Acids Res. *42*, 4160–4179.

Makarova, K.S., Anantharaman, V., Grishin, N.V., Koonin, E.V., and Aravind, L. (2014). CARF and WYL domains: ligand-binding regulators of prokaryotic defense systems. Front. Genet. *5*, 102.

Masaki, H., and Ogawa, T. (2002). The modes of action of colicins E5 and D, and related cytotoxic tRNases. Biochimie *84*, 433–438.

Matelska, D., Steczkiewicz, K., and Ginalski, K. (2017). Comprehensive classification of the PIN domain-like superfamily. Nucleic Acids Res. *45*, 6995–7020.

Michelini, F., Jalihal, A.P., Francia, S., Meers, C., Neeb, Z.T., Rossiello, F., Gioia, U., Aguado, J., Jones-Weinert, C., Luke, B., et al. (2018). From "cellular" RNA to "smart" RNA: multiple roles of RNA in genome stability and beyond. Chem. Rev. *118*, 4365–4403.

Miller, J.H. (1972). Experiments in Molecular Genetixs (Cold Spring Harbor Laboratory Press).

Molina, R., Stella, S., Feng, M., Sofos, N., Jauniskis, V., Pozdnyakova, I., López-Méndez, B., She, Q., and Montoya, G. (2019). Structure of Csx1-cOA$_4$ complex reveals the basis of RNA decay in Type III-B CRISPR-Cas. Nat. Commun. *10*, 4302.

Mueller, P.R., and Wold, B. (1989). In vivo footprinting of a muscle specific enhancer by ligation mediated PCR. Science *246*, 780–786.

Narumi, I., Satoh, K., Kikuchi, M., Funayama, T., Yanagisawa, T., Kobayashi, Y., Watanabe, H., and Yamamoto, K. (2001). The LexA protein from *Deinococcus radiodurans* is not involved in RecA induction following gamma irradiation. J. Bacteriol. *183*, 6951–6956.

Neu, H.C., and Heppel, L.A. (1964). Nucleotide sequence analysis of polyribonucleotides by means of periodate oxidation followed by cleavage with an amine. J. Biol. Chem. *239*, 2927–2934.

Niewoehner, O., Garcia-Doval, C., Rostøl, J.T., Berk, C., Schwede, F., Bigler, L., Hall, J., Marraffini, L.A., and Jinek, M. (2017). Type III CRISPR-Cas systems produce cyclic oligoadenylate second messengers. Nature *548*, 543–548.

O'Reilly, E.K., and Kreuzer, K.N. (2004). Isolation of SOS constitutive mutants of *Escherichia coli*. J. Bacteriol. *186*, 7149–7160.

Padan, E., Bibi, E., Ito, M., and Krulwich, T.A. (2005). Alkaline pH homeostasis in bacteria: new insights. Biochim. Biophys. Acta *1717*, 67–88.

Park, J.H., Yamaguchi, Y., and Inouye, M. (2012). Intramolecular regulation of the sequence-specific mRNA interferase activity of MazF fused to a MazE fragment with a linker cleavable by specific proteases. Appl. Environ. Microbiol. *78*, 3794–3799.

Ponting, C.P. (1997). P100, a transcriptional coactivator, is a human homologue of staphylococcal nuclease. Protein Sci. *6*, 459–463.

Popow, J., Englert, M., Weitzer, S., Schleiffer, A., Mierzwa, B., Mechtler, K., Trowitzsch, S., Will, C.L., Lührmann, R., Söll, D., and Martinez, J. (2011). HSPC117 is the essential subunit of a human tRNA splicing ligase complex. Science *331*, 760–764.

Potter, S.C., Luciani, A., Eddy, S.R., Park, Y., Lopez, R., and Finn, R.D. (2018). HMMER web server: 2018 update. Nucleic Acids Res. *46* (*W1*), W200–W204.

Rouillon, C., Athukoralage, J.S., Graham, S., Grüschow, S., and White, M.F. (2019). Investigation of the cyclic oligoadenylate signaling pathway of type III CRISPR systems. Methods Enzymol. *616*, 191–218.

Shames, S.R., Liu, L., Havey, J.C., Schofield, W.B., Goodman, A.L., and Roy, C.R. (2017). Multiple *Legionella pneumophila* effector virulence phenotypes revealed through high-throughput analysis of targeted mutant libraries. Proc. Natl. Acad. Sci. USA *114*, E10446–E10454.

Shiomi, D., Sakai, M., and Niki, H. (2008). Determination of bacterial rod shape by a novel cytoskeletal membrane protein. EMBO J. *27*, 3081–3091.

Singer, M.F. (1958). Phosphorolysis of oligoribonucleotides by polynucleotide phosphorylase. J. Biol. Chem. *232*, 211–228.

Tanaka, N., and Shuman, S. (2011). RtcB is the RNA ligase component of an *Escherichia coli* RNA repair operon. J. Biol. Chem. *286*, 7727–7731.

Tanaka, M., Earl, A.M., Howell, H.A., Park, M.J., Eisen, J.A., Peterson, S.N., and Battista, J.R. (2004). Analysis of *Deinococcus radiodurans*'s transcriptional response to ionizing radiation and desiccation reveals novel proteins that contribute to extreme radioresistance. Genetics *168*, 21–33.

Tanaka, N., Chakravarty, A.K., Maughan, B., and Shuman, S. (2011). Novel mechanism of RNA repair by RtcB via sequential 2′,3′-cyclic phosphodiesterase and 3′-phosphate/5′-hydroxyl ligation reactions. J. Biol. Chem. *286*, 43134–43143.

Tarn, W.-Y., Yario, T.A., and Steitz, J.A. (1995). U12 snRNA in vertebrates: Evolutionary conservation of 5' sequences implicated in splicing of pre-mRNAs containing a minor class of introns. RNA *1*, 644–656.

Temmel, H., Müller, C., Sauert, M., Vesper, O., Reiss, A., Popow, J., Martinez, J., and Moll, I. (2017). The RNA ligase RtcB reverses MazF-induced ribosome heterogeneity in *Escherichia coli*. Nucleic Acids Res. *45*, 4708–4721.

Trapnell, C., Hendrickson, D.G., Sauvageau, M., Goff, L., Rinn, J.L., and Pachter, L. (2013). Differential analysis of gene regulation at transcript resolution with RNA-seq. Nat. Biotechnol. *31*, 46–53.

Watanabe, H., Fauzi, H., Iwama, M., Onda, T., Ohgi, K., and Irie, M. (1995). Base non-specific acid ribonuclease from Irpex lacteus, primary structure and phylogenetic relationships in RNase T2 family enzyme. Biosci. Biotechnol. Biochem. *59*, 2097–2103.

Winther, K.S., and Gerdes, K. (2011). Enteric virulence associated protein VapC inhibits translation by cleavage of initiator tRNA. Proc. Natl. Acad. Sci. USA *108*, 7403–7407.

Wyatt, M.D., and Pittman, D.L. (2006). Methylating agents and DNA repair responses: methylated bases and sources of strand breaks. Chem. Res. Toxicol. *19*, 1580–1594.

Wyatt, H.D., and West, S.C. (2014). Holliday junction resolvases. Cold Spring Harb. Perspect. Biol. *6*, a023192.

Yang, W. (2011). Nucleases: diversity of structure, function and mechanism. Q. Rev. Biophys. *44*, 1–93.

Yang, M., Derbyshire, M.K., Yamashita, R.A., and Marchler-Bauer, A. (2020). NCBI's conserved domain database and tools for protein domain analysis. Curr. Protoc. Bioinformatics *69*, e90.

Zhang, D., Iyer, L.M., Burroughs, A.M., and Aravind, L. (2014). Resilience of biochemical activity in protein domains in the face of structural divergence. Curr. Opin. Struct. Biol. *26*, 92–103.

Zimmermann, L., Stephens, A., Nam, S.Z., Rau, D., Kübler, J., Lozajic, M., Gabler, F., Söding, J., Lupas, A.N., and Alva, V. (2018). A completely reimplemented mpi bioinformatics toolkit with a new HHpred server at its core. J. Mol. Biol. *430*, 2237–2243.

STAR★METHODS

KEY RESOURCES TABLE

REAGENT or RESOURCE	SOURCE	IDENTIFIER
Antibodies		
Monoclonal ANTI-FLAG M2 antibody produced in mouse	Sigma-Aldrich	Cat#F1804; RRID: AB_262044
HRP anti-*E. coli* RNA polymerase Sigma 70	BioLegend	Cat#663205; RRID: AB_2629596
Rabbit anti-*E. coli* RecA	Narumi et al., 2001	N/A
Rabbit anti-*E. coli* LexA	gifts of I. Narumi, Toyo University, Itakura, Gunma Japan	N/A
Rabbit anti-*E. coli* RtcB	Temmel et al., 2017	N/A
HA Tag Monoclonal Antibody, HRP	Thermo Fisher Scientific	Cat#26183; RRID: AB_2533056
Rabbit anti-*E. coli* RplE	gift of J. Zengel, University of Maryland, Baltimore County, MD	N/A
Rabbit anti-*E. coli* LexA	Abcam	Cat#ab174384; RRID: N/A
Rabbit IgG HRP Linked Whole Ab	Sigma-Aldrich	Cat#GENA934; RRID: AB_2722659
Goat anti-Mouse IgG Fc Cross-Adsorbed Secondary Antibody, HRP	Thermo Fisher Scientific	Cat#31439; RRID: AB_228292
Bacterial and Virus Strains		
List of Strains Used in This Study	This paper	See Table S3
Chemicals, Peptides, and Recombinant Proteins		
2,6-Diaminopimelic acid	Sigma-Aldrich	Cat#D1377; CAS: 583-93-7
Mitomycin C	Sigma-Aldrich	Cat#M4287; CAS: 50-07-7
Methyl methanesulfonate	Sigma-Aldrich	Cat#129925; CAS: 66-27-3
Bleomycin	Sigma-Aldrich	Cat#B8416; CAS: 9041-93-4
Phenol solution (acid)	Sigma-Aldrich	Cat#P4682; CAS: 108-95-2
X-gal	MP Biomedicals	Cat#114063102; CAS: 7240-90-6
DNAzol	Thermo Fisher Scientific	Cat#10503027
RNAprotect Bacteria Reagent	QIAGEN	Cat#76506
Turbo DNase I	Thermo Fisher Scientific	Cat#AM2238
Calf intestinal alkaline phosphatase	Sigma-Aldrich	Cat#11097075001
T4 polynucleotide kinase	New England Biolabs	Cat#M0201S
T4 RNA Ligase 2, truncated KQ	New England Biolabs	Cat#M0373S
Polyethylene glycol 8000	Sigma-Aldrich	Cat#1546605; CAS: 25322-68-2
RNaseOUT	Thermo Fisher Scientific	Cat#10777019
Superscript III Reverse Transcriptase	Thermo Fisher Scientific	Cat#18080093
Phusion DNA polymerase	New England Biolabs	Cat#M0530S
Factor Xa Protease	New England Biolabs	Cat#P8010S
ATP, [γ-^{32}P]	PerkinElmer	Cat#BLU002Z250UC
Chloramphenicol	Sigma-Aldrich	Cat#C0378; CAS 56-75-7
Carbenicillin	Sigma-Aldrich	Cat#C1389; CAS 4800-94-6
Tetracycline	Sigma-Aldrich	Cat#T3383; CAS 64-75-5
2-Mercaptoethanol	Sigma-Aldrich	Cat#M6250; CAS 60-24-2
2-Nitrophenyl-β-D-galactopyranoside	Sigma-Aldrich	Cat#N1127; CAS 369-07-3
Pierce ECL Western Blotting Substrate	Thermo Fisher Scientific	Cat#32106
SYBR Gold Nucleic Acid Gel Stain	Thermo Fisher Scientific	Cat#S11494

(*Continued on next page*)

Continued		
REAGENT or RESOURCE	SOURCE	IDENTIFIER
Critical Commercial Assays		
RNA Clean and Concentrator-5	Zymo Research	Cat#R1013
Zero Blunt PCR cloning kit	Thermo Fisher Scientific	Cat#K270020
TruSeq RNA Library Prep Kit v2	Illumina	Cat#RS-122-2001
Bacteria Ribo-Zero Magnetic Kit	Illumina	Cat#MRZB12424
Deposited Data		
Raw and Analyzed Data	This paper	GEO: GSE153782
Oligonucleotides		
List of Oligonucleotides Used in This Study.	This paper	See Table S3
Recombinant DNA		
List of Plasmids used in this study.	This paper	See Table S3
Software and Algorithms		
ImageJ	NIH	https://imagej.net/Welcome
GraphPad Prism 8.0	GraphPad	https://www.graphpad.com/scientific-software/prism/
Pfam	El-Gebali et al., 2019	N/A
CDD	Yang et al., 2020	N/A
HMMSCAN (HMMER3 package)	Potter et al., 2018	https://www.ebi.ac.uk/Tools/pfa/hmmer3_hmmscan/
PSI-BLAST	Altschul and Koonin, 1998	https://blast.ncbi.nlm.nih.gov/Blast.cgi?CMD=Web&PAGE=Proteins&PROGRAM=blastp&RUN_PSIBLAST=on
JACKHMMER	Johnson et al., 2010	https://www.ebi.ac.uk/Tools/hmmer/search/jackhmmer
HHPRed	Zimmermann et al., 2018	https://toolkit.tuebingen.mpg.de/tools/hhpred
Kalign program	Lassmann, 2019	N/A
Pymol	Schrödinger	https://pymol.org/2/
Illumina basecalling RTA 1.18.66.3	Illumina	N/A
Trimmomatic version 0.30 software	Bolger et al., 2014	http://www.usadellab.org/cms/?page=trimmomatic
Bowtie2 version 2.3.3.	Langmead and Salzberg, 2012	http://bowtie-bio.sourceforge.net/bowtie2/index.shtml
Cuffdiff	Trapnell et al., 2013	http://cole-trapnell-lab.github.io/cufflinks/cuffdiff/
Other		
Amersham Hybond-N	Cytiva	Cat#RPN303N
Ni-NTA Agarose	Thermo Fisher Scientific	Cat#R90101
Superdex 200 Increase 10/300 Column	Cytiva	Cat#28990944
Amicon Ultra-15 Centrifugal Filter Units	Millipore	Cat#UFC901024
Whatman Optitran Nitrocellulose Blotting Membrane	Cytiva	Cat# 10439196

RESOURCE AVAILABILITY

Lead Contact

Further information and requests for resources and reagents should be directed to and will be fulfilled by the Lead Contact, Sandra Wolin (sandra.wolin@nih.gov).

Materials Availability

Bacterial strains and plasmids are available on request.

CellPress
OPEN ACCESS

Data and Code Availability

The accession number for the RNA sequencing data reported in this paper is Gene Expression Omnibus: GSE153782.

EXPERIMENTAL MODEL AND SUBJECT DETAILS

All bacterial strains used in this study are listed in Table S3. Two virulent *S.* Typhimurium strains were used: SL1344 (Hoiseth and Stocker, 1981) (gift of Jorge Galan, Yale School of Medicine) and 14028s (Jarvik et al., 2010) (gift of Eduardo Groisman, Yale School of Medicine). Mutant strains were created using allelic exchange (Kaniga et al., 1994) using the donor strain *E. coli* β-2163Δnic35 and R6K-based conjugative transfer of suicide vectors (Demarre et al., 2005). To generate deletion mutants, DNA fragments 5′ and 3′ to the ORF were amplified from genomic DNA using primers p1 and p2r, and p3 and p4r, respectively (Table S3), which were joined with overlap PCR. To generate the RtcR mutant, which encodes a truncated protein containing 185 amino acids at the N terminus, DNA fragments were amplified from genomic DNA using 5′-ATCCTTCATCATCCCGCGTTCG-3′ and 5′-GTATTAAAACGCACGCTGTCGCCTGCCGGACTTCAGGAAGTTGAG-3′, and 5′-GGCGACAGCGTGCGTTTTAATAC-3′ and 5′-GATCTTAACGATCTGGCGGAACAG-3′ and joined by overlap PCR. DNAs were cloned into the SmaI site of pSB890 and transformed into donor strain *E. coli* β-2163Δnic35, which was used to conjugate recipient strains. P22 phage transductants were constructed as described (Davis et al., 1980). All strains were grown at 37° in Luria-Bertani broth (LB; 10 g/L tryptone, 10 g/L NaCl, 5 g/L yeast extract) with appropriate antibiotic unless otherwise stated. Chloramphenicol was used at 15 μg/ml, carbenicillin was used at 100 μg/ml, tetracycline was used at 12-20 μg/ml, and diaminopimelic acid (DAP) was used at 50 μg/ml. For treatment with DNA damaging agents, cells were grown in LB to OD_{600} = 0.9 and incubated with 3 μM (1 μg/ml) MMC (Sigma-Aldrich), 0.02% methyl methanesulfonate (MMS) (Sigma) or 1 or 5 μg/ml bleomycin for 2 h unless otherwise stated. Bacteria were collected and resuspended in RNAprotect Bacteria reagent (QIAGEN). Afterward, bacteria were centrifuged and RNA extracted using hot acid phenol (Chen et al., 2007). *E. coli* strain MG1655 was a gift of Donald Court (National Cancer Institute, Frederick, MD, USA).

METHOD DETAILS

Transposon mutagenesis

Plasmid pSRS_CM1 (Shames et al., 2017), also called pSB4807 (Fowler and Galan, 2018), a chloramphenicol-resistant derivative of pSAM_Bt (Goodman et al., 2009), was a gift of C. Fowler and J. Galan. This plasmid was transferred into donor strain *E. coli* β-2163Δnic35 (Demarre et al., 2005) and propagated at 37°C in LB containing DAP and carbenicillin. Conjugative-based transposon mutagenesis of recipient *S.* Typhimurium strains was performed by mixing donor and recipient strains in a 3:1 ratio. Bacterial conjugations were performed at room temperature for 24 h and transconjugants selected by plating on LB agar plates containing 15 mg/ml chloramphenicol and 40 mg/ml X-gal (MP Biomedicals) to identify blue colonies. Approximately 63,000 colonies were screened (28,000 14028s and 35,000 SL1344) and blue colonies purified by streaking to single colonies on X-gal/chloramphenicol plates. Genomic DNA was isolated with DNAzol (Thermo Fisher), partially digested with Sau3AI and ligated to a DNA adaptor digested with BamHI. Ligation-mediated PCR (Mueller and Wold, 1989), using primers that anneal to adaptor and transposon sequences, was used to amplify sites of transposon insertion from genomic DNA.

β-Galactosidase assays

All strains were grown at 37°C in LB with the appropriate antibiotic. Expression of rtcR and rtcRΔN was induced from pBAD24 with 0% and 0.1% arabinose respectively. (Some product is produced in 0% arabinose because the promoter is leaky). β-galactosidase activity was measured as described (Miller, 1972) from cells grown to OD_{600} between 0.320 and 1.160 (with most strains kept below 1.0) unless otherwise stated. Briefly, after cultures were incubated on ice for 20 minutes to stop growth, cells were pelleted and re-suspended in Z buffer (60 mM $Na_2HPO_4.7H_2O$, 40 mM $NaH_2PO_4.H_2O$, 10 mM KCl, 1 mM $MgSO_4$, 50 mM 2-mercaptoethanol, adjusted to pH 7.0). Cells were diluted in Z buffer to 1 mL and permeabilized by adding 100 μl chloroform and 50 μl 0.1% sodium dodecyl sulfate and vortexing. After incubating at 28°C for 5 minutes, 200 μl 2-nitrophenyl-β-D-galactopyranoside (Sigma-Aldrich) was added. After yellow color developed, the reaction was stopped by adding 500 μl 1 M Na_2CO_3 and 1 mL transferred to a micro-centrifuge tube and the tube sedimented at 16,000 x g for 5 minutes. After measuring the OD420 and OD550 of the supernatant, units of activity were calculated according to the equation Miller units = 1000 x [(OD_{420} - 1.75 x OD_{550})] / [reaction time (minutes) x volume of culture (ml) x OD_{600} of original culture].

Construction of expression plasmids

The pRtcRΔN plasmid for overexpressing N-terminally truncated RtcR was described (Chen et al., 2013). To construct pRtcR, full-length RtcR was amplified from genomic DNA using 5′-GGAATTCATGCGAAAAACGGTGGCCTTTG-3′and 5′-GCTCTAGATTAATTCTGTAAAACGTCCCACGTCAG-3′, digested with EcoRI and XbaI and cloned into pBAD24. To construct pHA-RtcA (in which the HA epitope is fused to RtcA), full-length RtcA was amplified from genomic DNA with 5′-GGAATTCATGTACCCATACGATGTTCCAGATTACGCTATGGCAAGGATCATCGCGCTG-3′ and 5′-GCTCTAGATTAGTCGCTTACCCGGACAAGATAGC-3′, digested with EcoRI and XbaI and cloned into pBAD24.

Immunoblotting

Bacterial pellets were resuspended in 1X SDS sample buffer (50 mM Tris-HCl pH 6.8, 2% SDS, 10% glycerol, 100 mM DTT, 12.5 mM EDTA, 0.02% bromophenol blue). Cells were lysed by boiling for 10 min. After centrifuging at 16,000 x g, cleared lysates were fractionated in SDS-polyacrylamide gels and transferred to nitrocellulose. Membranes were blocked overnight in 5% milk in TBS-T (20 mM Tris-HCl pH 7.6, 137 mM NaCl, 0.1% Tween 20) overnight at 4°C, washed twice with TBS-T at room temperature, and incubated with primary antibody diluted in 5% milk in TBS-T at room temperature for 1 hour. Membranes were washed in TBS-T before incubation with HRP-coupled secondary antibody diluted in 5% milk in TBS-T for 1 hour at room temperature followed by washing with TBS-T. HRP was detected by enhanced chemiluminescence (ECL) using the Pierce ECL Western Blotting Substrate (ThermoFisher). Antibodies used for immunoblotting are listed in the Key Resources Table. In Figure S3K, the anti-LexA antibody was purchased from Abcam, while in Figure 4A, the anti-LexA antibody was a gift of Dr. I. Narumi (Toyo University).

RNA analyses and Northern blotting

Total RNA was extracted from bacterial pellets treated with RNAprotect Bacteria Reagent (QIAGEN) using hot acid phenol. Pellets (3 O.D. units) were resuspended in 400 μL of 10 mM Tris-HCl (pH 7.5), 10 mM EDTA, 0.5% SDS and 400 μL of acid phenol (pH 5) and incubated at 65°C for 30 minutes with occasional vortexing. The sample was then cooled briefly on ice, sedimented at 25°C for 10 minutes at 16,000 x g and the supernatant removed to a new microcentrofuge tube. After extraction with phenol:chloroform: isoamyl alcohol (50:49:1), RNA was precipitated with 2.5 volumes ethanol in 40 μL 3M NaOAc.

For 3′ end analyses, 10 μg of RNA was incubated in 5 μl 10X Turbo DNase I buffer (Ambion) and 2 U Turbo DNase I (Ambion) at 37°C for 30 minutes followed by purification using RNA Clean and Concentrator-5 (Zymo Research Corporation). To remove 3′ phosphates, RNA was first incubated with 10 mM HCl (Sigma) on ice for 4 hours, followed by incubation with 5 U of CIP (Roche), or 10 U T4 PNK (New England Biolabs) according to the manufacturers' instructions and purified using RNA Clean and Concentrator-5 kit (Zymo Research Corporation). Afterward, RNA (1.5 μg) was ligated overnight at 16°C to 750 ng of adenylated adaptor (Table S3) using T4 RNA Ligase 2, truncated KQ (New England Biolabs) in the presence of 25% PEG 8000 and RNaseOUT (Thermo Fisher). Ligated RNAs were extracted with phenol:chloroform:isoamyl alcohol (50:49:1), precipitated with ethanol, and resuspended in 10 μl water. Half the sample was used for Northern analyses, while the other half was subjected to reverse transcription using Superscript III (Thermo Fisher) and a primer complementary to the adenylated adaptor (Table S3). After reverse transcription, cDNA was amplified using Phusion DNA polymerase (New England Biolabs), a tRNA-specific forward primer (Table S3), and the RT-adaptor reverse primer. Gel purified DNAs were cloned using Zero Blunt PCR cloning kit (Thermo Fisher) according to manufacturer instructions. At least 9 clones were sequenced for each sample. For Northern analyses, RNA was separated in 5% or 8% polyacrylamide/8.3 M urea gels and transferred to Hybond N (Cytiva) in 0.5X TBE at 150 mA for 16 h. Blots were hybridized with [^{32}P]-labeled oligonucleotides as described (Tarn et al., 1995). Oligonucleotide probes are listed in Table S3. Radioactive signals were detected using a Typhoon FLA 7000 Phosphorimager (GE Healthcare).

RNA sequencing and analysis

Salmonella RNA was isolated from 14028s cells treated with or without MMC for two hours using hot acid phenol. The RNA library was prepared by the National Cancer Institute Sequencing Facility using the TruSeq RNA Library Prep Kit v2 (Illumina) following ribosomal RNA removal using the Ribo-Zero Magnetic Kit for bacteria (Illumina). Sequencing was performed on a NextSeq 500 (Illumina) with 75 bp paired end reads. After trimming adapters and low quality sequence using Trimmomatic version 0.30 software, reads were aligned to the *S*. Typhimirium (14028s) genome using Bowtie2 software version 2.3.2. Mapped BAM files were used as input for Cuffdiff to determine differential gene expression between MMC-treated and control untreated samples.

Genome screening for candidate tRNA endoribonucleases

All proteins encoded by the genomes of *S*. Typhimurium SL1344 and 14028s strains were screened against a library of profiles constructed from Pfam (El-Gebali et al., 2019), CDD (Yang et al., 2020) and a custom database of profiles of RNase domains from diverse biological conflict systems using the RPSBLAST and HMMSCAN program (HMMER3 package). Statistically significant hits ($e < 10^{-3}$) were checked for recovery of known and predicted endoribonuclease domains and these were set aside as potential candidates; for example, proteins containing domains that were members of the BECR fold (Zhang et al., 2014; Iyer et al., 2017), PIN domain superfamily (Matelska et al., 2017), SNase fold (Ponting, 1997), HEPN domain (Anantharaman et al., 2013), potential RNA endonucleases of the RNase H fold (Majorek et al., 2014) and metallo-beta-lactamase fold (Aravind, 1999), the RNase T2 domain (Watanabe et al., 1995), the RNaseE/G superfamily (Callaghan et al., 2005), and the SymE domain (Kawano et al., 2007) were considered valid hits. Proteins encoded by the two *S*. Typhimurium genomes were also subject to iterative sequence profile searches using PSI-BLAST (Altschul and Koonin, 1998) and JACKHMMER (Johnson et al., 2010) and profile-profile searches using the HHPRed program (Zimmermann et al., 2018) in a further effort to unify them with known domains. Successful unification with any of the known RNase domains led to their inclusion in the candidate list. Genome contexts of all candidates were then isolated and screened for 1) links to known translation or ribosome assembly factors and 2) inclusion in biological conflict systems. Active site conservation patterns of each candidate were examined for potential loss of catalytic activity using multiple alignments [constructed with the Kalign program (Lassmann, 2019) and, if required, examination of homologous structures using Pymol (https://pymol.org/2/)]. Predicted inactive representatives of the above RNase domains were removed from further consideration. We arrived at a list of 40 potential RNases. To

prioritize, whole transcriptome sequencing of rRNA-depleted total RNA from wild-type cells treated with or without MMC was performed. Potential RNases that were expressed in the presence of MMC were prioritized for deletion.

Survival assays

Overnight cultures containing 100 μg/ml carbenicillin were diluted to OD_{600} = 0.05 in LB containing 0.1% glucose, which reduces expression of genes controlled by the P_{BAD} promoter (Guzman et al., 1995). After 2.5 h at 37°, bacteria were diluted to OD_{600} = 0.2 in LB containing 0.1% glucose and 1 μg/ml MMC. After 2 additional h at 37°, bacteria were diluted and spotted or spread on LB agar plates. Colonies from both MMC- and mock-treated bacteria were counted to determine the fraction of surviving cells.

MazEF purification and MazF activation

To overexpress MazEF in *E. coli*, double-stranded DNA encoding the 41 C-terminal coding residues of the MazE antitoxin, a Factor Xa cleavage site and the coding sequence of the MazF toxin as a fusion protein (Park et al., 2012) was synthesized (gBlocks Gene Fragments, Integrated DNA Technologies), digested with NdeI and XhoI and inserted into pET28b (EMD Biosciences). After transforming the recombinant plasmid into *E. coli* BL21(DE3), cells were grown to OD_{600} = 0.8, induced with 0.5 mM IPTG at 25°C for 4 h and harvested by centrifugation. After resuspending in 20 mM Tris-HCl pH 7.5, 300 mM NaCl, 20 mM imidazole, cells were lysed by passing through a French Press at 8000 psi three times. After sedimenting the lysate at 40,000 rpm in a Beckman Type 50.2 Ti rotor, the supernatant was passed through a Ni-NTA agarose column (Thermo Fisher), washed with 20 mM Tris-HCl pH 7.5, 300 mM NaCl, 30 mM imidazole until the OD_{600} of the flowthrough was below 0.01. His-tagged MazEF was eluted with 20 mM Tris-HCl pH 7.5, 300 mM NaCl, 250 mM imidazole. Further purification was by size exclusion chromatography using a Superdex 200 Increase 10/300 column (GE Healthcare Life Sciences) in 20 mM Tris-HCl pH 7.5, 150 mM NaCl, 10% glycerol. Fractions containing MazEF were pooled, concentrated using Amicon Ultra-15 Centrifugal Filter Units (Millipore), aliquoted and frozen in liquid nitrogen. MazF was activated by incubating 1 mg MazEF with 10 μg protease Factor Xa (NEB) in 10 mM Tris-HCl pH 8.0, 1 mM DTT, 2 mM $CaCl_2$ at 37°C for 3 h (Rouillon et al., 2019).

Generation of 5′ tRNA halves for binding studies

RNA oligonucleotides corresponding to 5′ tRNA halves containing 3′-OH or 3′-phosphate ends were purchased from Integrated DNA Technologies. To generate RNAs containing 2′, 3′-cyclic phosphate, activated MazF was incubated with RNA oligonucleotides containing a MazF cleavage site ACACUG at the 3′ end (Rouillon et al., 2019). For EMSAs, RNAs were were labeled at the 5′ end using [γ-^{32}P]-ATP and T4 PNK. After incubating at 70°C for 20 min to inactivate T4 PNK, activated MazF was added to 10 μg/μl and incubated for 2 h at 37°C to generate RNAs ending in 2′, 3′-cyclic phosphate. To remove the cyclic phosphate and generate a labeled RNA with 3′-OH, the reaction was incubated with T4 PNK (Amitsur et al., 1987). To generate labeled RNA ending with 3′-phosphate, the desired sequence was synthesized with an additional uridylate, labeled at the 5′ end with [γ-^{32}P]-ATP and T4 PNK, and incubated in 100 μl 1 M DL-lysine-Cl and 0.025 M $NaIO_4$ (pH 8.3) at 45°C for 2.5 h (Neu and Heppel, 1964). Afterward, the RNA was extracted with phenol:chloroform:isoamyl alcohol (50:49:1) and precipitated with 2.5 volumes ethanol. All labeled RNAs were purified from 15% polyacrylamide, 8.3M urea gels before use.

Purification of full-length RtcR and the CARF domain

Sequences encoding full-length *S.* Typhimurium RtcR and the CARF domain (amino acids 1-188) were amplified from genomic DNA using primers 5′-CGGGATCCGATGCGAAAAACGGTGGCCTTTG-3′ and 5′-CCGCTCGAGTTAATTCTGTAAAACGTCCCACGTCAG-3′, and primers 5′-CGGGATCCGATGCGAAAAACGGTGGCCTTTG-3′ and 5′-CCGCTCGAGTTAGGTTGCAAT GCCGGACTTCAG-3′, respectively, digested with BamHI and XhoI and cloned into the same sites of pRSFDuet-1 (Novagen). The resulting plasmids were transformed into *E. coli* BL21(DE3). To express recombinant protein, cells were cultured in LB containing 20 mM Tris-HCl pH 7.5 and 50 μg/ml kanamycin to OD_{600} = 0.7, 0.1mM IPTG was added and the culture incubated at 16°C for 20 h. His-tagged proteins were purified as described above, except that the lysis buffer was 50 mM Tris-HCl pH 7.5, 500 mM NaCl, 10% glycerol, 20 mM imidazole, the wash buffer was 50 mM Tris-HCl pH 7.5, 500 mM NaCl, 10% glycerol, 30 mM imidazole, the elution buffer was 50 mM Tris-HCl pH 7.5, 500 mM NaCl, 10% glycerol, 250 mM imidazole and the buffer used in gel filtration was 20 mM Tris-HCl pH 7.5, 250 mM NaCl, and 10% glycerol.

RNA binding assays

^{32}P-labeled RNAs in binding buffer (20 mM Tris-HCl pH 7.5, 50 mM NaCl, 1 mM $MgCl_2$, 0.05% Tween 20) were refolded by heating to 95°C for 2 min, frozen on dry ice and thawed on ice. Refolded RNAs were mixed with RtcR or the CARF domain in binding buffer in 5 μl total volume, incubated at 4°C for 30 min and at room temperature for 30 min. For RtcR, the reaction included 1 mM ATP except where stated. Reactions were fractionated in 6% polyacrylamide (80:1 acrylamide:bisacrylamide)/5% glycerol gels for CARF domain EMSAs and 4% polyacrylamide (80:1 acrylamide:bisacrylamide)/2.5% glycerol gels for RtcR EMSAs. Gels were run at 4°C, 5 V/cm for 20 min, then 10 V/cm in 0.5xTBE (50 mM Tris, 45 mM boric acid, 1.25 mM EDTA) until the bromophenol blue dye migrated 4 cm. The gels were dried and scanned using a Typhoon FLA 7000 Phosphorimager (GE Healthcare Life Sciences). Fractions of bound RNA were quantitated using ImageJ (NIH). Results were analyzed with GraphPad Prism 8 and fitted by nonlinear regression using the equation for one site specific binding: $Y = Bmax*X/(Kd + X)$, where Y is the fraction of bound RNA, Bmax is the maximum specific binding, X is the protein concentration, and *K*d is the equilibrium binding constant.

Size exclusion chromatography

Size exclusion chromatography was performed on an ÄKTA Pure 25 using a Superdex 200 Increase 10/300 column, which was equilibrated and run in 20 mM Tris-HCl pH 7.5, 100 mM NaCl, 1 mM $MgCl_2$, 2% glycerol, 0.5% Tween 20, 1 mM ATP, with a flow rate at 0.25 ml/min. Next, 48 μM of RtcR and 48 μM of the refolded RNA were mixed in gel filtration buffer. After incubating 30 min on ice and at room temperature for 30 min to allow complex formation, 100 μl of the sample was injected onto the column. Elution volumes were monitored by measuring the absorbance at 280 nm. Proteins in peak fractions were analyzed using 4%–12% SDS-PAGE and staining with Coomassie blue, while RNAs was detected by fractionation in 8% polyacrylamide/8.3 M urea gels, followed by staining with SYBR Gold Nucleic Acid Gel Stain (Thermo Fisher).

QUANTIFICATION AND STATISTICAL ANALYSIS

Statistical analysis

All results are presented as the mean (n = 3) of three biological replicates ± SEM. GraphPad Prism v8 was used for statistical analysis. P values were calculated using two-tailed unpaired t tests. *, $p < 0.05$; **, $p < 0.01$, ***, $p < 0.001$ indicate significant differences between samples; ns indicates not significant.

Cell Reports, Volume *33*

Supplemental Information

An RNA Repair Operon
Regulated by Damaged tRNAs

Kevin J. Hughes, Xinguo Chen, A. Maxwell Burroughs, L. Aravind, and Sandra L. Wolin

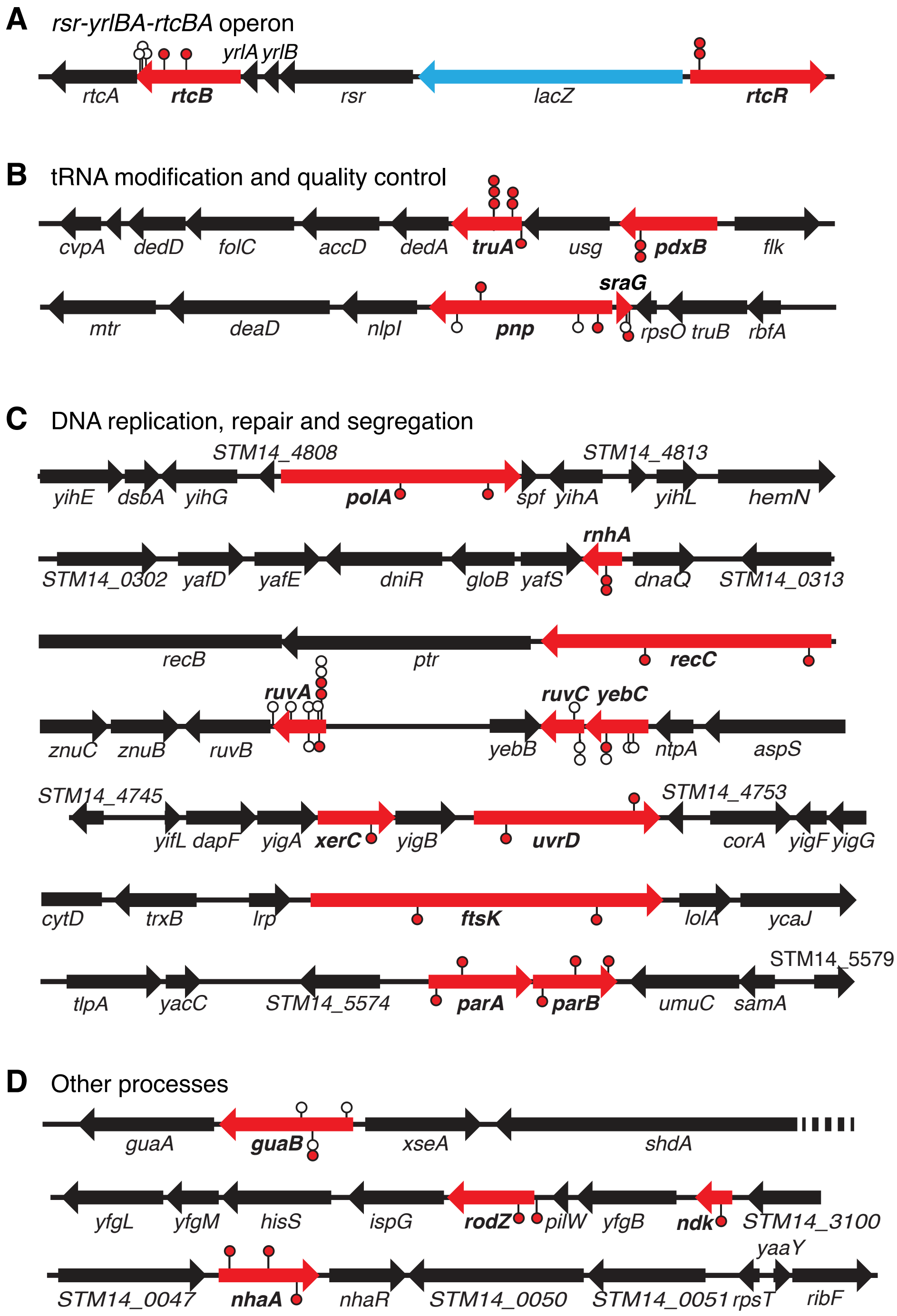

Figure S1, related to Figure 1. Location and orientation of transposon insertions resulting in P_{rsr}-lacZ expression.

(A-D) Transposon insertion sites in *S.* Typhimurium 14028s (red circles) and SL1344 (white circles) strains are indicated. Insertions in the Watson strand are shown above each locus and insertions in the Crick strand are shown below. Loci containing at least two independent insertions are shown, together with *xerC* and *ndk*, which are upstream from genes containing multiple insertions. Loci not described in the main text are *pdxB*, which encodes erythronate-4-phosphate dehyrogenate, which functions in the synthesis of pyridoxal 5′-phosphate, *xerC*, a site-specific DNA recombinase required for chromosome segregation (Blakely et al., 1993) (panel C), *guaB*, which encodes the inosine monophosphate dehydrogenase that functions in guanine biosynthesis, *ndk*, which encodes nucleotide diphosphate kinase, *rodZ*, a transmembrane protein required for the *E. coli* rod shape (Shiomi et al., 2008) and *nhaA*, which encodes the N+/H+ antiporter that maintains intracellular pH (Padan et al., 2005) (all panel D). The insertion in *pdxB* could have a polar effect on *truA* transcription.

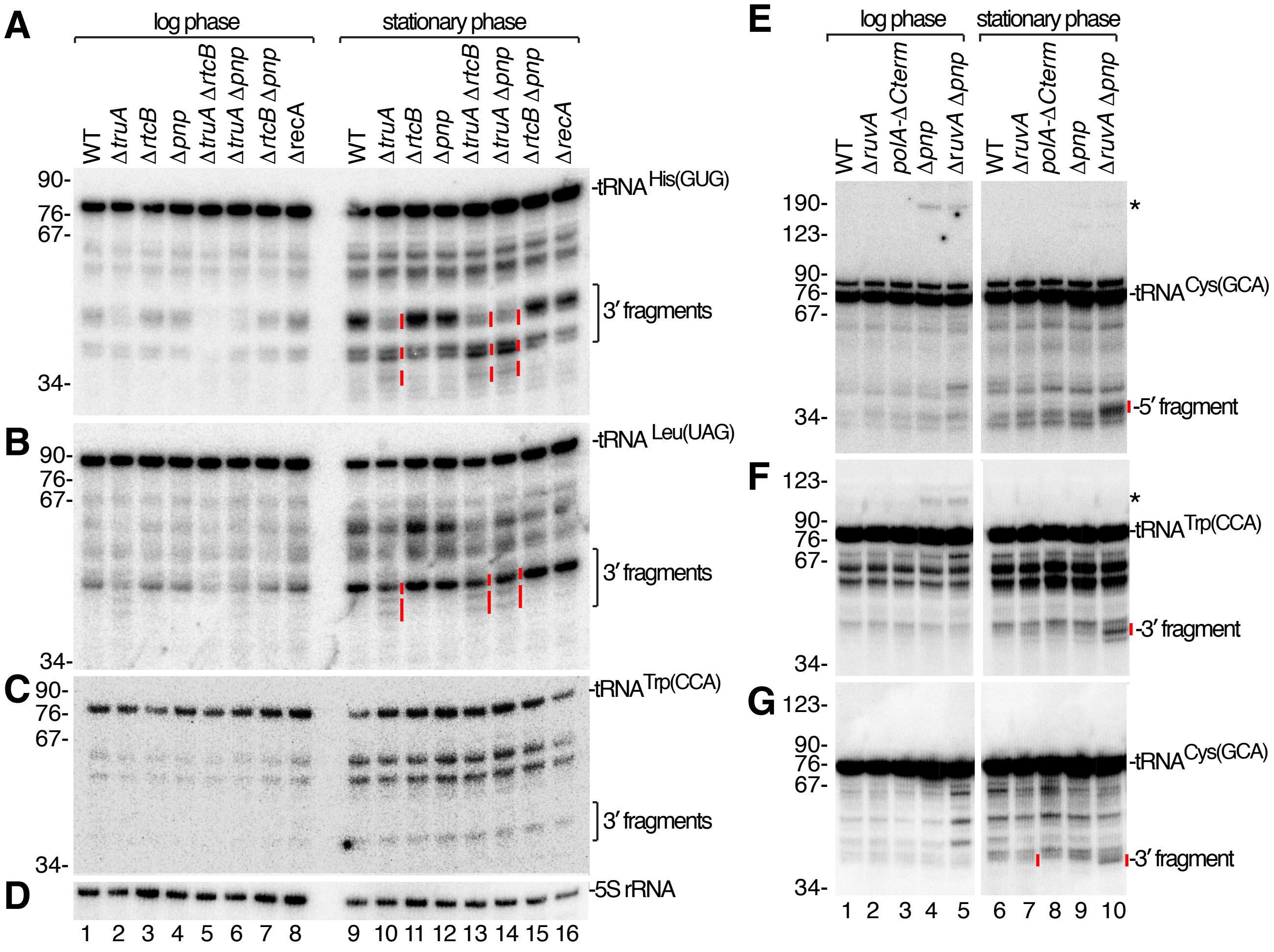

Figure S2, related to Figure 2. Accumulation of tRNA fragments in mutant strains.

(A-D) Following growth to mid-log (lanes 1-8) or stationary phase (lanes 9-16), RNA was extracted from the indicated strains and subjected to Northern blotting. Blots were probed to detect 3' halves of $tRNA^{His(GUG)}$ (A), $tRNA^{Leu(UAG)}$ (B) and $tRNA^{Trp(CCA)}$ (C). Fragments that differ in mobility or levels in Δ*truA* strains grown in stationary phase are indicated with red lines. As a loading control, the blot was reprobed to detect 5S rRNA (D).

(E-G) RNA from the indicated strains grown to mid-log (lanes 1-5) or stationary phase (lanes 6-10) was subjected to Northern blotting to detect 5' halves of $tRNA^{Cys(GCA)}$ (E) and 3' halves of $tRNA^{Trp(CCA)}$ (F) and $tRNA^{Cys(GCA)}$ (G). The asterisks denote tRNA precursors that accumulate in Δ*pnp* strains. Fragments differing in mobility or levels in mutant strains grown in stationary phase are indicated with red lines.

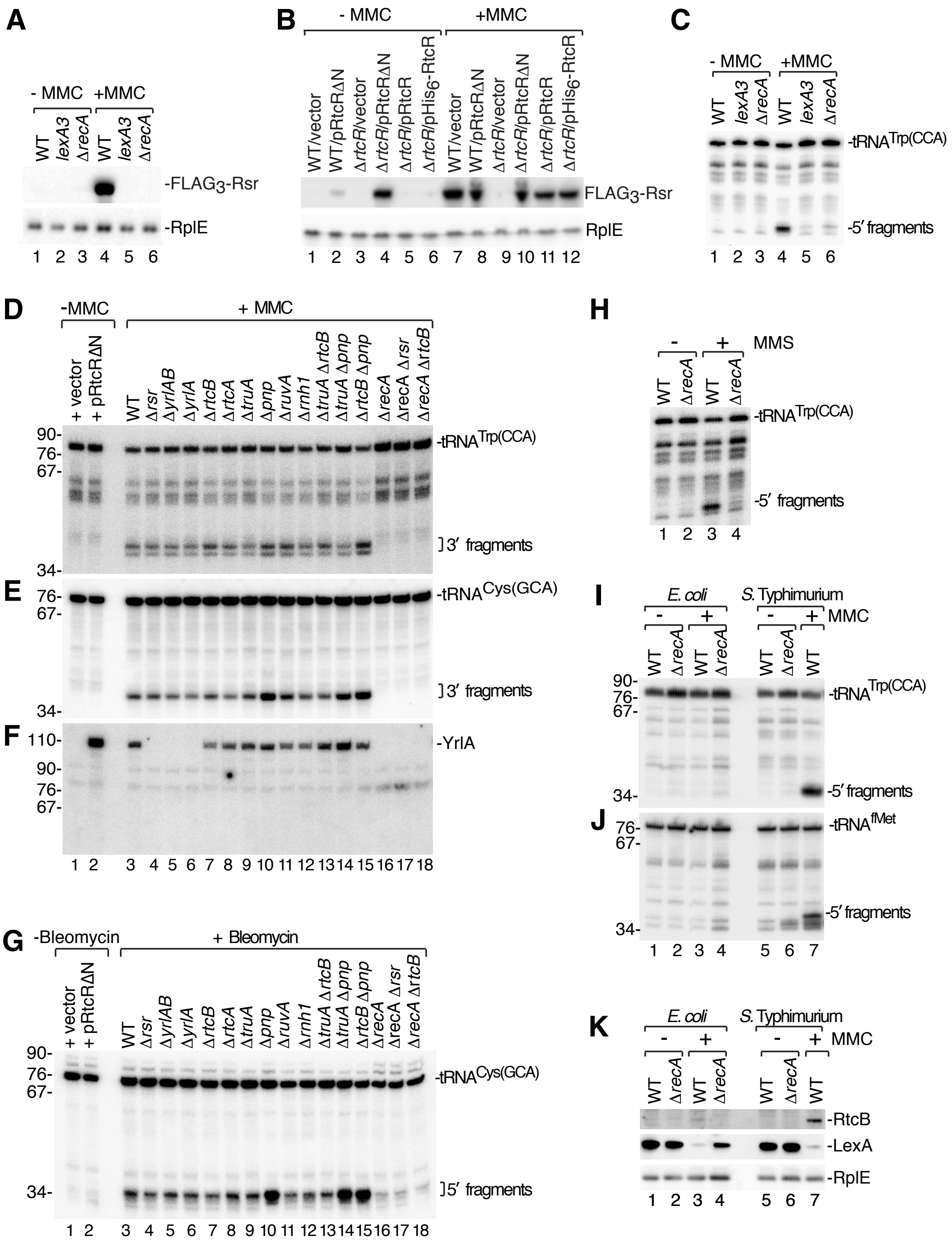

Figure S3, related to Figure 4. DNA damage results in tRNA cleavage and operon induction

(A) After treating wild-type, Δ*recA* and *lexA3* strains carrying *FLAG3-rsr* without (lanes 1-3) or with MMC (lanes 4-6) for 2 hours, lysates were immunoblotted to detect $FLAG_3$-Rsr and RplE (loading control).

(B) Wild-type (lanes 1, 2, 7, 8) and Δ*rtcR* strains (lanes 3-6 and 9-12) carrying *FLAG3-rsr* were grown without (lanes 1-6) or with MMC (lanes 7-12) and the resulting lysates subjected to Western blotting to detect $FLAG_3$-Rsr and Rpl5. To confirm that lack of operon expression was due to loss of RtcR, Δ*rtcR* strains carrying plasmids expressing RtcR (lanes 5, 11) and His_6-RtcR (lanes 6, 12) were examined. Strains carrying pRtcRΔN were also assayed (lanes 4, 10).

(C) After treating the strains without (A) (lanes 1-3) or with MMC (lanes 4-6) for 2 hours, RNA was extracted and subjected to Northern blotting to detect $tRNA^{Trp(CCA)}$ 5' fragments.

(D-F) After two hours in MMC, RNA was extracted from the indicated strains and subjected to Northern analysis to detect 3' fragments of $tRNA^{Trp(CCA)}$ (D), $tRNA^{Cys(GCA)}$ (E) or 5' fragments of YrlA RNA (F) (lanes 3-18). As a control, RNA from wild-type cells carrying the pRtcRΔN plasmid or an empty vector was assayed (lanes 1-2).

(G) After growing the indicated strains to mid-log, bleomycin was added and the strains incubated for 2 hours at 37°C. RNA was subjected to Northern analysis to detect 5' fragments of $tRNA^{Cys(GCA)}$ (lanes 3-18). Lanes 1-2, RNA from wild-type cells carrying either an empty vector or the pRtcRΔN plasmid.

(H) After treating wild-type and Δ*recA FLAG$_3$-rsr* strains either without (lanes 1-2) or with MMS (lanes 3-4) for 2 hours, RNA was subjected to Northern blotting to detect 5' halves of $tRNA^{Trp(CCA)}$.

(I and J) After growing *E. coli* MG1655 and *S*. Typhimurium 14028s strains with or without MMC, RNA was probed to detect 5' fragments of $tRNA^{Trp(CCA)}$ (I) and $tRNA^{fMet}$ (J).

(K) After growth with or without MMC for two hours, lysates of the indicated *E. coli* and *S*. Typhimurium strains were subjected to immunoblotting to detect RtcB, LexA and RplE.

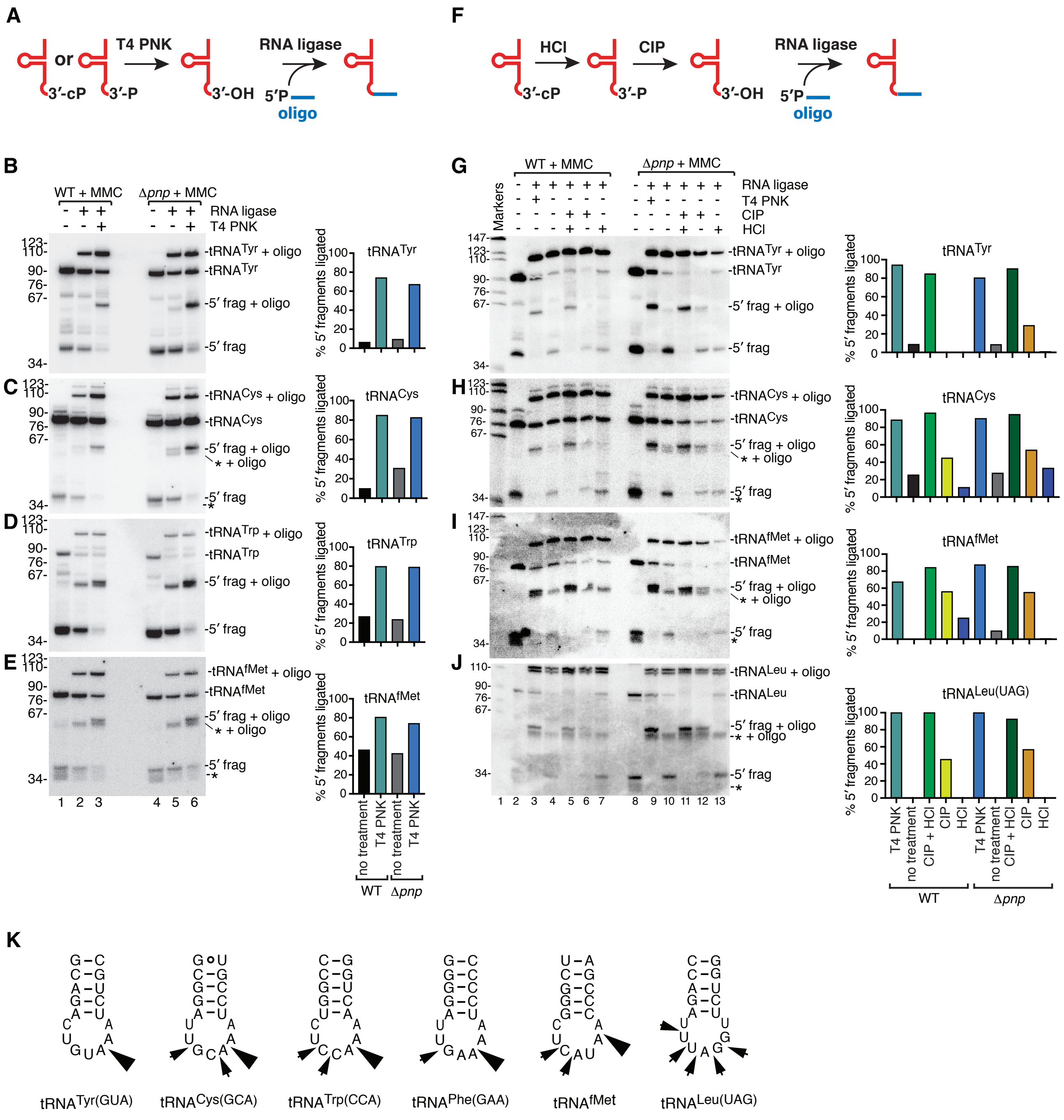

Figure S4, related to Figure 5. tRNA 5' fragments end in cyclic phosphate

(A) Schematic of strategy used to distinguish 5' fragments ending in 2', 3'-cyclic phosphate and 3'-phosphate from 5' fragments ending in 3'-OH.

(B-E) Left panels: After growing wild-type (lanes 1-3) and Δ*pnp* strains (lanes 4-6) with MMC for two hours, RNA was extracted and incubated without (lanes 1, 2, 4, 5) or with (lanes 3, 6) T4 PNK prior to adding T4 RNA ligase and a 5'-phosphate-containing oligonucleotide. After gel fractionation, Northern blotting was used to detect $tRNA^{Tyr(GUA)}$ (B), $tRNA^{Cys(GCA)}$ (C), $tRNA^{Trp(CCA)}$ (D) and $tRNA^{fMet}$ (E). Right panels: Quantitation. The asterisks in C and E denote shorter fragments of $tRNA^{Cys(GCA)}$ and $tRNA^{fMet}$ that end in 3'-OH.

(F) Schematic of strategy used to distinguish 5' fragments ending in 2', 3'-cyclic phosphate from 5' fragments ending in 3'-phosphate.

(G-J) To distinguish between 3'-phosphate and 2', 3'-cyclic phosphate, RNA from MMC-treated wild-type (lanes 2-7) and Δ*pnp* strains (lanes 8-13) was incubated with acid (HCl) and calf-intestinal phosphatase (CIP, lanes 5 and 11) or CIP alone (lanes 6 and 12), prior to adding T4 RNA ligase and the 5'-phosphate containing oligonucleotide. Northern blotting was used to detect $tRNA^{Tyr(GUA)}$ (G), $tRNA^{Cys(GCA)}$ (H), $tRNA^{fMet}$ (I) and $tRNA^{Leu(UAG)}$ (J). As a positive control, T4 PNK was added in place of CIP (lanes 3 and 9). As negative controls, the indicated components were omitted from the reactions (lanes 2, 4, 7, 8, 10, 13). Lane 1, size markers. Right panels: Quantitation. The asterisks in H, I and J denote shorter fragments of $tRNA^{Cys(GCA)}$, $tRNA^{fMet}$ and $tRNA^{Leu(UAG)}$ that end in 3'-OH.

(K) Cleavage sites of $tRNA^{Tyr(GUA)}$, $tRNA^{Cys(GCA)}$, $tRNA^{Trp(CCA)}$, $tRNA^{Phe(GAA)}$, $tRNA^{fMet}$ and $tRNA^{Leu(UAG)}$ determined by 3' RACE. For each tRNA, at least 9 cDNAs were sequenced (see Table S2). Arrowheads denote 3' ends present in more than half the sequences, while small arrows denote minor 3' ends.

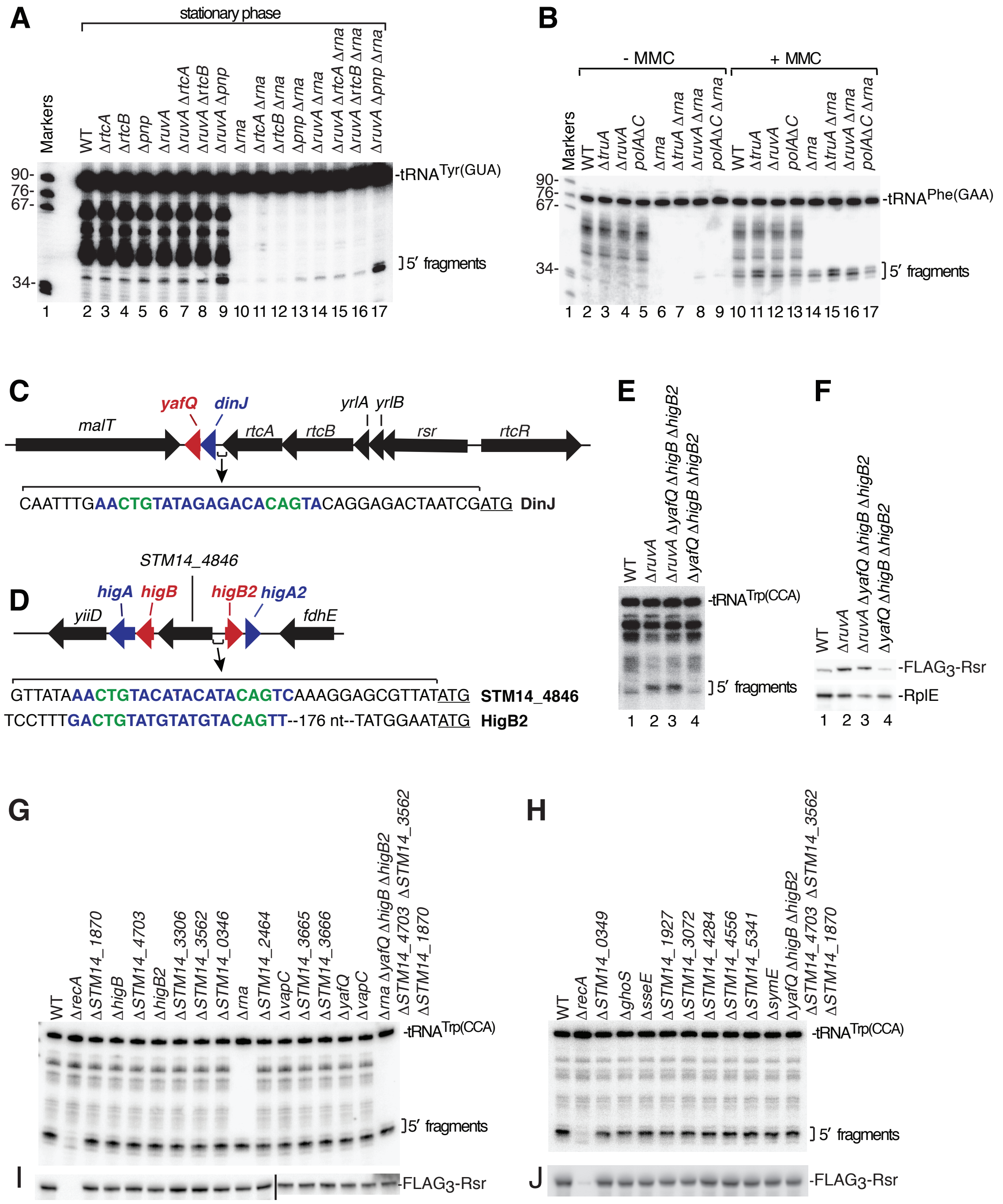

Figure S5, related to Figure 5. Deletion of multiple candidate endonucleases does not affect tRNA cleavage or operon activation

(A) RNA from the experiment shown in Figures 5A-5C was subjected to Northern blotting to detect 5' fragments of $tRNA^{Tyr\ (GUA)}$.

(B) RNA from the experiment shown in Figures 5D-G was subjected to Northern blotting to detect 5' fragments of $tRNA^{Phe(GAA)}$.

(C and D) Genetic map of the *dinJ-yafQ* (C) and *higB-higA* and *higB2-higA2* (D) neighborhoods. The sequences of the putative LexA sites are shown.

(E) RNA extracted from the indicated strains was subjected to Northern blotting to detect 5' fragments of $tRNA^{Trp(CCA)}$.

(F) Lysates of the strains in (E) were subjected to immunoblotting to detect $FLAG_3$-Rsr and RplE (loading control).

(G and H) After growing for two hours in MMC, RNA was isolated from the indicated strains and subjected to Northern blotting to detect $tRNA^{Trp(CCA)}$ 5' fragments. In the last lanes of (G) and (H), RNA was extracted from strains lacking seven (G) or six (H) potential nucleases.

(I and J) After two hours of growth in MMC, lysates were prepared from the strains shown in (G and H) and immunoblotting was performed to detect $FLAG_3$-Rsr.

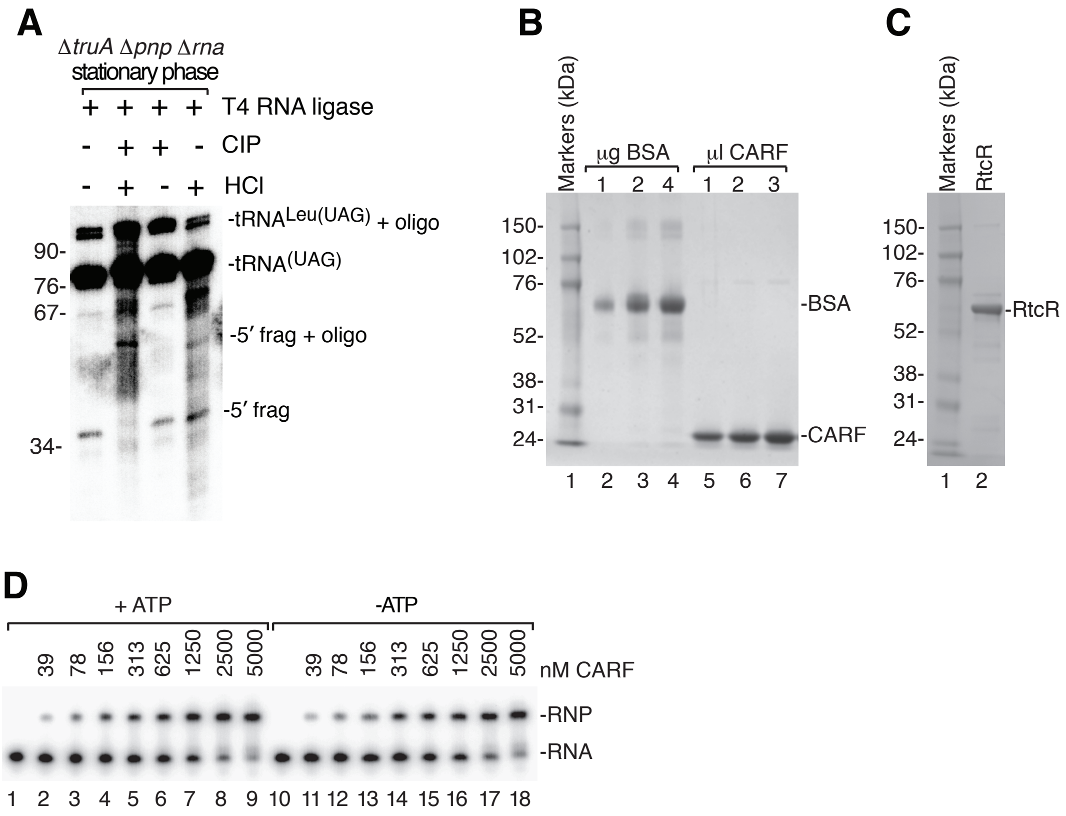

Figure S6, related to Figure 6. Binding of 5' tRNA halves to the RtcR CARF domain

(A) To determine whether the 5' tRNA$^{Leu(UAG)}$ fragments that accumulate in Δ*truA* Δ*pnp* Δ*rna* strains in stationary phase ended with 3'-OH, 3'-phosphate, or 2', 3'-cyclic phosphate, total RNA from these cells was incubated with acid (HCl) and or calf intestinal phosphatase (CIP) prior to adding T4 RNA ligase and the 5'-phosphate containing oligonucleotide. RNA in lane 1 received no acid or CIP. Northern blotting was used to detect tRNA$^{Leu(UAG)}$.

(B) To quantitate the purified protein, increasing amounts of CARF domain were subjected to SDS-PAGE together with 1, 2 and 4 μg of purified bovine serum albumin and visualized by Coomassie blue staining.

(C) Purified RtcR (1 μg) was subjected to SDS-PAGE and stained with Coomassie blue.

(D) To examine whether binding to the isolated CARF domain was dependent on ATP, 5' tRNA$^{Leu(UAG)}$ halves ending in cyclic phosphate were mixed with the indicated concentrations of the isolated CARF domain with or without 1 mM ATP.

Table S2. 3' ends of tRNA 5' halves that accumulate in MMC (determined by 3' RACE), related to Figures 5 and S4.

tRNA$^{Cys(GCA)}$	
**GCGGATTGCAAATCCGTCTAGTCCGGTTCGACTCCGGAACGCGCCTCCA**	
GCGGATTGCA	(8)
GCGGATTGC	(1)
GCGGATT	(3)
tRNA$^{Trp(CCA)}$	
**CCGGTCTCCAAAACCGGGTGTTGGGAGTTCGAGTCTCTCCGCCCCTGCCA**	
CCGGTCTCCA	(6)
CCGGTCTC	(2)
CCGGTCT	(1)
tRNA$^{Tyr(GUA)}$	
**GCAGACTGTAAATCTGCCGTCATCGACTTCGAAGGTTCGAATCCTTCCCCCACCACCA**	
GCAGACTGTA	(12)
tRNA$^{Phe(GAA)}$	
**GGGGATTGAAAATCCCCGTGTCCTTGGTTCGATTCCGAGTCCGGGCACCA**	
GGGGATTGAA	(10)
GGGGATT	(2)
tRNA$^{(fMet)}$	
**TCGGGCTCATAACCCGAAGGTCGTCGGTTCAAATCCGGCCCCCGCAACCA**	
TCGGGCTCATA	(8)
TCGGGCTC	(4)
TCGGGCT	(2)
tRNA$^{Leu(UAG)}$	
**CCAGATTTAGGTTCTGGCGCCGCAAGGTGTGCGAGTTCAAGTCTCGCCTCCCGCACCA**	
CCAGATTTAG	(1)
CCAGATTTA	(4)
CCAGATTT	(3)
CCAGATT	(5)
CCAGAT	(2)

Sequences of clones obtained from 3' RACE. The number in parentheses denotes the number of independent clones for each sequence. A portion of the full length tRNA sequence is at the top in bold and the anticodon is underlined.
